# Association between dried fruit intake and pan-cancers incidence risk: A two-sample Mendelian randomization study

**DOI:** 10.3389/fnut.2022.899137

**Published:** 2022-07-18

**Authors:** Chen Jin, Rui Li, Tuo Deng, Zixia Lin, Haoqi Li, Yan Yang, Qing Su, Jingxian Wang, Yi Yang, Juejin Wang, Gang Chen, Yi Wang

**Affiliations:** ^1^Department of Epidemiology and Biostatistics, School of Public Health and Management, Wenzhou Medical University, Wenzhou, China; ^2^Department of Oncology, Tongji Hospital, Huazhong University of Science and Technology, Wuhan, China; ^3^Department of Hepatobiliary Surgery, The First Affiliated Hospital of Wenzhou Medical University, Wenzhou, China; ^4^Key Laboratory of Diagnosis and Treatment of Severe Hepato-Pancreatic Diseases of Zhejiang Province, The First Affiliated Hospital of Wenzhou Medical University, Wenzhou, China

**Keywords:** dried fruit intake, site-specific cancers, causal relationship, Mendelian randomization, incidence risk

## Abstract

**Background:**

Observational studies have revealed that dried fruit intake may be associated with cancer incidence; however, confounding factors make the results prone to be disturbed. Therefore, we conducted a two-sample Mendelian randomization (MR) study to explore the causal relationship between dried fruit intake and 11 site-specific cancers.

**Materials and methods:**

Forty-three single nucleoside polymers (SNPs) with robust genome-wide association study (GWAS) evidence, strongly correlated with dried fruit intake, were used as instrumental variables (IVs) in this study. The summary-level genetic datasets of site-specific cancers were obtained from the Oncoarray oral cavity and oropharyngeal cancer consortium, International Lung Cancer Consortium, Breast Cancer Association Consortium (BCAC), Ovarian Cancer Association Consortium, PanScan1, and GWAS of other scholars. We analyzed the causality between dried fruit intake and 11 site-specific cancers using the inverse-variance-weighted (IVW) and weighted median (WM) methods. For the results of the MR analysis, Cochran’s *Q* test was used to check for heterogeneity, and multiplicative random effects were used to evaluate the heterogeneity further. Gene pleiotropy was tested using MR-Egger regression and MR-PRESSO methods. In addition, the main results of this study were validated by using the summary statistical data from the FinnGen and UK Biobank databases, and adjusted body mass index (BMI), years of education, fresh fruit intake, and vitamin C using multivariable MR analysis to ensure the stability of the research results.

**Results:**

The evidence from IVW analyses showed that each increase of dried fruit intake by one standard deviation was statistically significantly associated with 82.68% decrease of oral cavity/pharyngeal cancer incidence risk (*P* = 0.0131), 67.01% decrease of lung cancer incidence risk (*P* = 0.0011), 77% decrease of squamous cell lung cancer incidence risk (*P* = 0.0026), 53.07% decrease of breast cancer incidence risk (*P* = 4.62 × 10^–5^), 39.72% decrease of ovarian cancer incidence risk (*P* = 0.0183), 97.26% decrease of pancreatic cancer incidence risk (*P* = 0.0280), 0.53% decrease of cervical cancer incidence risk (*P* = 0.0482); however, there was no significant effect on lung adenocarcinoma (*P* = 0.4343), endometrial cancer (*P* = 0.8742), thyroid cancer (*P* = 0.6352), prostate cancer (*P* = 0.5354), bladder cancer (*P* = 0.8996), and brain cancer (*P* = 0.8164). In the validation part of the study results, the causal relationship between dried fruit intake and lung cancer (*P* = 0.0043), squamous cell lung cancer (*P* = 0.0136), and breast cancer (*P* = 0.0192) was determined. After adjusting for the potential impact of confounders, the causal relationship between dried fruit intake and lung cancer (*P* = 0.0034), squamous cell lung cancer (*P* = 0.046), and breast cancer (*P* = 0.0001) remained. The sensitivity analysis showed that our results were stable and reliable.

**Conclusion:**

The intake of dried fruits may have a protective effect against some site-specific cancers. Therefore, health education and a reasonable adjustment of dietary proportions may help in the primary prevention of cancer.

## Introduction

Cancer is a major global health problem and the second leading cause of morbidity and mortality, resulting in a heavy disease burden (1). In recent years, significant progress has been made in cancer treatment (2), early detection (3, 4), and control of specific risk factors, such as smoking (5), polycyclic aromatic hydrocarbon (6), cyclophosphamide (7), and carcinogenic infection (8, 9); however, the harm of cancer to human health and quality of life still exists. Generally, cancer prevention focuses on specific risk factors, such as tobacco use, diet, living habits, and carcinogen infection, which is determined by its high complexity and heterogeneity (10). Studies have shown that more than 30% of cancers are caused by dietary factors (11, 12). Therefore, adjusting dietary patterns and changing dietary habits can effectively prevent cancer development.

Traditional observational epidemiological studies have shown that tumor incidence is associated with insufficient intake of fruits and vegetables. For instance, increased dietary fiber consumption may have additional benefits in patients with colorectal cancer after diagnosis (13). Fruits and vegetables are considered protective factors in the etiology of lung cancer, even though the confounding effects of smoking cannot be ruled out (14). The risk of prostate cancer is significantly reduced when the intake of fruits and vegetables is high (15). However, most observational studies on the relationship between diet and cancer do not distinguish between dried fruit and raw fruit or do not mention the impact of dried fruit on cancer. Dietary guidelines in many countries also encourage people to choose non-juice-form fruits as much as possible, including dried fruit (16). Dried fruit is a stable form of fruit that remains fresh through drying technology; however, it mainly appears in the human diet as a snack, accounting for a relatively small proportion. Some clinical and laboratory studies have reported that the intake of dried fruit is related to the progression or occurrence of some cancers. Nevertheless, the discussion on the relationship between dried fruit intake and the risk of cancer is only based on animal models or laboratory data (17, 18), and there is a lack of reliable epidemiological causality assessments.

Traditional observational studies may lead to deviation and even misjudgment of the research results due to various observable and unobservable residual confounding factors, reverse causality, and bias; meanwhile, they mainly focus on the correlation between exposure factors and outcomes rather than the actual causal relationship. Mendelian randomization (MR) is a new epidemiological method that imitates the design of randomized controlled studies (19). It uses single nucleotide polymorphisms (SNPs) as instrumental variables (IVs) to infer causality between the risk factors and outcomes of interest. SNP is randomly assigned to individuals with gametes during meiosis (20), which is similar to the requirements of randomized controlled trials. Simultaneously, genetic variation precedes the occurrence of diseases, which avoids the potential impact of reverse causality.

Therefore, MR is an ideal method to explore the causal relationship between dried fruit intake and pan-cancer. This study used a two-sample MR design to investigate whether dried fruit intake has a causal relationship with 11 site-specific cancers and estimate its effect to provide scientific evidence for cancer primary prevention.

## Materials and methods

### Study design

**FIGURE 1:**
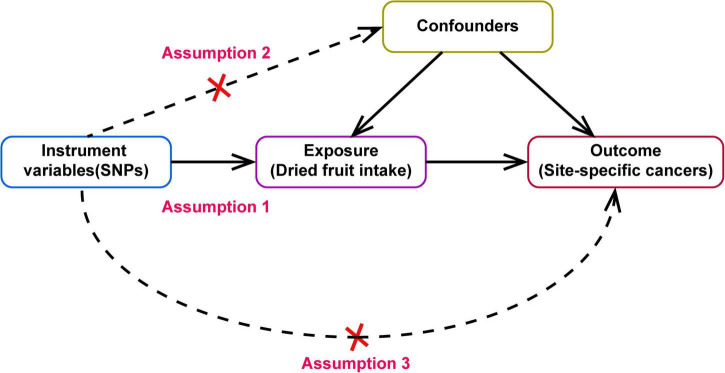
Directed acyclic graph of Mendelian randomization (MR) framework showing the hypothesis of dried fruit intake on site-specific cancers, the dotted line indicates that there has pleiotropic or direct causal relationship between exposure and outcome.

A two-sample MR design was used to evaluate the causal effect of dried fruit intake on cancer risk (Figure 1). The MR design is based on the following three core assumptions: First, genetic IVs must be closely related to dried fruit intake (Assumption 2). Second, confounding factors cannot affect the selected IVs that influence the association between dried fruit intake and cancer (Assumption 1). Third, IVs can only affect cancer risk through dried fruit intake (Assumption 3).

### Dried fruit intake exposure data source

The genome-wide association study (GWAS) summary statistics of dried fruit intake were obtained from the UK Biobank, which is a large cohort of approximately 500,000 individuals aimed at collecting the genotype and various phenotypic data and was approved by the Research Ethics Committee (21, 22) (REC reference is 11/NW/0382). All participants in the cohort were invited to the local evaluation center to obtain corresponding data using a touch-screen questionnaire or anthropometry with standardized procedures. Dried fruit intake, as an exposure factor was obtained by questioning the frequency of dried fruit intake in the questionnaire. Participants were asked, “how many pieces of dried fruit would you eat per day?” (Count one prune, one dried apricot, and ten raisins as one piece; put “0” if you do not eat any). Answer with the average (integer) of participants’ intake in the past year. Other options are “10,” “1,” or “3,” representing less than one, do not know, and prefer not to answer, respectively. Finally, 421,764 participants of European ancestry obtained dried fruit intake as the exposure factor through the questionnaire’s frequency of dried fruit intake.

### Site-specific cancers outcome data sources

We considered 11 site-specific cancers as the outcomes of this study (lung cancer selected three datasets: lung cancer, lung adenocarcinoma, and squamous cell lung cancer). The sources and corresponding information for all the aggregated statistical datasets used in this study are listed in Supplementary Table 1.

The GWAS summary statistics of oral/pharyngeal cancer were obtained from the Oncoarray oral cavity and oropharyngeal cancer consortium, included 2,342 cases and 2,329 controls mainly from the International Head and Neck Cancer Epidemiology Consortium (INHANCE), as well as a European cohort study (EPIC) and the United Kingdom case-series (HN5000) (23); GWAS summary data for lung cancer (27,209 participants including 11,348 patients and 15,861 controls) squamous cell lung cancer (18,313 participants with 3,275 cases and 15,038 controls) and lung adenocarcinoma (18,336 participants with 3,442 cases and 14,894 controls) were from the International Lung Cancer Association (ILCCO), and the patient data were based on previously reported GWAS: IARC-GWAS, NCI-GWAS, ICR-GWAS, and MDACC-GWAS (24–27); the GWAS summary data of breast cancer on 33,832 participants (15,748 breast cancer patients and 18,084 controls) came from the Breast Cancer Association Consortium (BCAC), included 8 (C-BCAC) and a subset of BPC3 GWAS (CGEMS) (28–31); participants in epithelial ovarian cancer were from the Ovarian Cancer Associations Consortium (OCAC, including 25,509 population-based patients and 40,941 controls) (32); the pancreatic cancer summary data of PanScan1 consortium included 1,896 cases and 1,939 controls, this is a GWAS based on 12 prospective cohort (33); the summary statistics of endometrial cancer were obtained in meta-GWAS of O’Mara et al. (34), including 12,906 cases of endometrial cancer and 108,979 country-matched controls; the controls were from 17 studies of the Endometrial Cancer Association Consortium (ECAC), the Epidemiology of Endometrial Cancer Consortium (E2C2), and the UK Biobank; the summary data of thyroid cancer were from Kohler et al. (35), a GWAS based on 649 patients with thyroid cancer and 431 controls; the summary data of prostate cancer generated in GWAS by Schumacher et al. (36), including 79,148 patients and 61,106 controls; the summary statistics for bladder cancer included 1,279 patients and 372,016 controls; summary statistics for brain cancer included 606 patients and 372,016 controls; and summary statistics for cervical cancer included 563 patients and 198,523 controls.

All of the above cancer datasets were acquired from individuals of European ancestry. All consortiums obtained informed consent from the participants and the approval of the relevant ethics committees when participants participated in the study.

### Selection of instrumental variables

To explore the causal relationship between dried fruit intake and site-specific cancers better, SNPs were used as IVs. The criteria selected for SNPs were as follows: (i) the SNPs were highly correlated with dried fruit intake, which is significant for whole-genome research, that is, *P* < 5 × 10^–8^. (ii) SNPs are independent of each other to avoid offset caused by linkage disequilibrium (LD). When *R*^2^ of the LD was greater than 0.001, one of them was eliminated. (iii) Genetic distance refers to the length of the region, considering LD. Here, we set the genetic distance as 10,000 kb; within 10,000 kb, remove the SNP with *R*^2^ greater than 0.001 with the most significant SNPs. SNPs characteristics of dried fruit intake were extracted, including SNP number, chromosome location, effective allele, effective allele frequency (EAF), effect value, standard error, and *P*-value of the effective allele and dried fruit intake.

The *F*-statistic of each SNP was used to judge the correlation strength and avoid bias caused by weak IVs to ensure a strong correlation between IVs and exposure factors. When the *F* value was greater than 10, it was considered that there was no bias in weak IVs. The following formula was used to calculate the *F*-statistic for each SNP:


$$F=\frac{N-K-1}{K}\times \frac{R^{2}}{1-R^{2}}$$

where *N* is the sample size of the exposure dataset, *K* is the number of SNPs, and *R*^2^ represents the proportion of variation explained by IVs in the exposure dataset; specifically, the calculation formula of *R*^2^ is:


(1) R=22×(1-MAF)×MAF×(βSD)2

here, MAF is the secondary allele frequency, equivalent to EAF when calculating *R*^2^, β is the allele effect value, and *SD* is the standard deviation.

In addition, IVs identified for inclusion in this study were searched on the PhenoScanner website^1^ to detect pleiotropic effects for the selected IVs, if there was any SNP correlated with the outcomes, they should be excluded from the IVs prior to perform MR analysis. The detailed results of the search are presented in Supplementary Table 2. We found that IVs were strongly associated with body mass index (BMI) and years of education; therefore, these two factors were also included in the multivariate MR analysis to exclude their effects on the causal relationship between exposure and outcome.

### Univariate two-sample Mendelian randomization analysis

This study used two MR methods to estimate the relationship between dried fruit intake and cancer: inverse-variance-weighted (IVW) and weighted median (WM). The premise of the IVW method is that all the IVs are effective. If any SNP does not meet the assumption of IV, the result will be biased. Multiple SNPs can enhance the statistical ability of MR analysis. However, due to the existence of pleiotropy, when some SNPs do not meet the hypothesis of IV, the causal relationship between dried fruit intake and cancers will deviate. However, when 50% of SNPs are effective IV, the estimation obtained by the WM method should be consistent with the actual effect (37, 38).

The intercept term of the MR-Egger regression model was used to test whether there was gene pleiotropy. If the intercept term was close to 0 (*P* < 0.05), it was considered that the influence of genetic pleiotropy was small. IVs were not directly related to outcome events, then Assumption 3 is valid. The MR-Pleiotropy RESidual Sum and Outlier (MR-PRESSO) method, which corrects the estimate by eliminating outliers, was also used to detect the existence of gene pleiotropy. Cochran’s *Q* test was used to assess IV heterogeneity to evaluate further the impact of heterogeneity on causal estimation. When heterogeneity was present in the results, a multiple random effects model was used to re-estimate causality. Simultaneously, SNPs are removed one by one, and the remaining SNPs continue to be analyzed by MR, using the leave-one-out analysis method to investigate the sensitivity of the results.

### Multivariate Mendelian randomization analysis

According to the results of the search on the PhenoScanner website and possible confounding factors between dried fruit intake and outcomes (fresh fruit intake and vitamin C), we used a multivariate MR analysis with the addition of fresh fruit intake, vitamin C, BMI, and years of education to adjust for causal effects between exposure and outcome in five rounds of adjustment: (i) fresh fruit intake alone, (ii) vitamin C alone, (iii) BMI alone, (iv) years of education alone, and (v) a combination of fresh fruit intake, vitamin C, BMI, and years of education.

### Mendelian randomization in validation datasets

To validate the main findings, 12 cancer datasets from the FinnGen database and two cancer datasets from the UK Biobank database (endometrial cancer and oral cavity/pharyngeal cancer were not available in the FinnGen database, and summary statistics from the UK Biobank database were used) were used as outcomes for the two-sample MR analysis. As the summary statistics for dried fruit intake were also obtained from the UK Biobank database, the results of the IVW method were corrected for endometrial cancer and oral cavity/pharyngeal cancer using the *MRlap* function to avoid any possible overlap of samples affecting the causality. The sources of all datasets used in the validation are listed in Supplementary Table 1.

### Statistical analyses

All analyses were performed using R software (version 4.0.5) under the Windows environment. The R packages used for all MR-related analyses and image plotting included “*vcfR*,” “*TwoSampleMR*,” “*MR-PRESSO*,” “*MRlap*,” and “*forestplot*.” A two-sided *P* < 0.05 was considered a statistically significant difference.

## Results

### Details of instrumental variables

**FIGURE 2:**
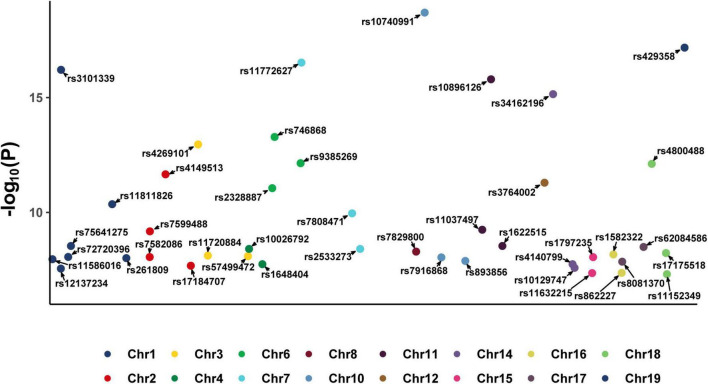
Manhattan plot of 43 SNPs identified as instrumental variables (IVs) from exposure dataset. SNP, single nucleotide polymorphism.

After screening, 43 SNPs that were closely related to dried fruit intake (*P* < 5 × 10^–8^) and independent of each other (*R*^2^ < 0.001) were identified (Supplementary Table 3). A Manhattan plot of these 43 SNPs is shown in Figure 2. The average *F*-statistic was 24.7464 (range, 17.4989–47.9013), indicating that the results are less likely to deviate owing the influence of weak IVs, consistent with Assumption 1. Based on the search results on the PhenoScanner website, there were no cancer-associated SNPs among these 43 IVs; therefore, we used these 43 SNPs as IVs to estimate the causal effects of dried fruit intake and 11 site-specific cancers in the subsequent analysis.

### Causal effect analysis between dried fruit intake and site-specific cancers

**FIGURE 3:**
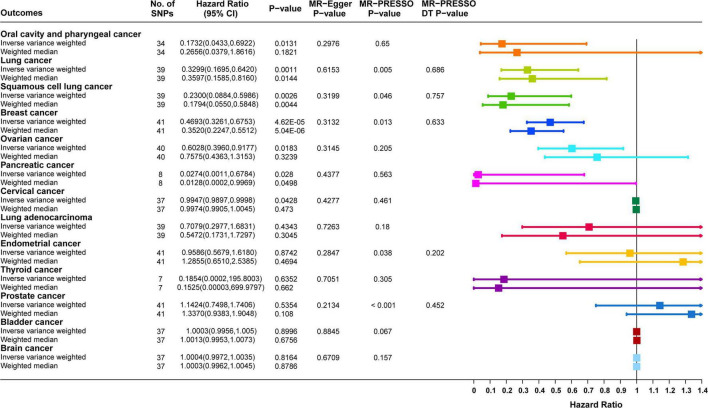
Forest plot of two-sample Mendelian randomization (MR) estimation of the association between dried fruit intake and cancer risk. No. of SNPs, number of single nucleotide polymorphisms; CI, confidence interval.

Causal correlation analysis of dried fruit intake and 11 site-specific cancers used inverse variance weighting (IVW) and weight median (WM) methods. The results of the IVW method supported the causal relationship between dried fruit intake and oral cavity/pharyngeal, lung, squamous cell lung, breast, ovarian, pancreatic, and cervical cancers. The higher the dried fruit intake, the lower the cancer incidence risk. The risk of oral cavity/pharyngeal cancer decreased by 82.68% (OR = 0.1732, 95% CI: 0.0433–0.6922, *P* = 0.0131) for every increase of dried fruit intake by one standard deviation; lung cancer risk was reduced by 67.01% (OR = 0.3299, 95% CI: 0.1695–0.642, *P* = 0.0011); the risk of squamous cell lung cancer was reduced by 77.00% (OR = 0.2300, 95% CI: 0.0884–0.5986, *P* = 0.0026); the risk of breast cancer was reduced by 53.07% (OR = 0.4693, 95% CI: 0.3261–0.6753, *P* = 4.62 × 10^–5^); the risk of ovarian cancer was reduced by 39.72% (OR = 0.6028, 95% CI: 0.3960–0.9177, *P* = 0.0183); the risk of pancreatic cancer was reduced by 97.26% (OR = 0.0274, 95% CI: 0.0011–0.6784, *P* = 0.0280); the risk of cervical cancer was reduced by 0.53% (OR = 0.9947, 95% CI: 0.9897–0.9998, *P* = 0.0482). The WM method also supported a causal relationship between dried fruit intake and lung, squamous cell lung, breast, and pancreatic cancers. However, for lung adenocarcinoma, endometrial, thyroid, prostate, bladder, and brain cancers, neither the IVW nor WM method showed statistical significance. The details of the results are presented in Figure 3 and Table 1.

**TABLE 1:** Two-sample Mendelian randomization (MR) analyses of the association between dried fruit intake and eleven site-specific cancers.

Outcome	IVW method			WM method		
	OR	95% CI of OR	*P*-value	OR	95% CI of OR	*P*-value
Oral cavity and pharyngeal cancer	0.1732	(0.0433, 0.6922)	0.0131*	0.2656	(0.0379, 1.8616)	0.1821
Lung cancer	0.3299	(0.1695, 0.6420)	0.0011*	0.3597	(0.1585, 0.8160)	0.0144*
Squamous cell lung cancer	0.2300	(0.0884, 0.5986)	0.0026*	0.1794	(0.0550, 0.5848)	0.0044*
Breast cancer	0.4693	(0.3261, 0.6753)	4.62 × 10^–5^*	0.3520	(0.2247, 0.5512)	5.04 × 10^–6^*
Ovarian cancer	0.6028	(0.3960, 0.9177)	0.0183*	0.7575	(0.4363, 1.3153)	0.3239
Pancreatic cancer	0.0274	(0.0011, 0.6784)	0.0280*	0.0128	(0.0002, 0.9969)	0.0498*
Cervical cancer	0.9947	(0.9897,0.9998)	0.0428*	0.3045	(0.1731, 1.7297)	0.3045
Lung adenocarcinoma	0.7079	(0.2976, 1.6831)	0.4342	1.2855	(0.6510, 2.5385)	0.4694
Endometrial cancer	0.9586	(0.5679, 1.6180)	0.8742	0.1525	(3.32 × 10^–5^, 699.9797)	0.6620
Thyroid cancer	0.1854	(0.0001, 195.8003)	0.6352	1.3370	(0.9383, 1.9048)	0.1080
Prostate cancer	1.1424	(0.7498, 1.7406)	0.5354	1.3370	(0.9383, 1.9048)	0.1080
Bladder cancer	1.0003	(0.9956,1.0050)	0.8996	1.0013	(0.9953,1.0073)	0.6756
Brain cancer	1.0003	(0.9972,1.0035)	0.8164	1.0003	(0.9961,1.0045)	0.8786

OR, odds ratios; 95% CI, 95% confidence interval; IVW, inverse-variance-weighted; WM, weighted median.

*Indicate P < 0.05. The bold values represent the statistically significant P-values.

The scatter plots show the estimated effect of IVs on exposure and outcomes, and the rising slope in the plot indicates a negative correlation between dried fruit intake and the risk of site-specific cancer (Supplementary Figure 1). In addition, because of the results extracted from different outcome datasets and the deletion of palindrome SNPs with intermediate allele frequencies, the number of IVs used in the causal analysis between dried fruit intake and various types of cancer was not equal.

The funnel plot (Supplementary Figure 2) shows that when a single SNP was used as an IV, the causal effects were symmetrically distributed, indicating that the results were less likely to be affected by potential bias and that the results were stable and reliable.

### Sensitivity analysis on results of univariate two-sample Mendelian randomization

The existence of gene pleiotropy was tested using MR-Egger regression analysis. Among the 11 site-specific cancers (oral cavity/pharyngeal, lung, squamous cell lung, breast, ovarian, cervical, lung adenocarcinoma, pancreatic, endometrial, thyroid, prostate, bladder, and brain), all of the intercept term was close to zero (*P* > 0.05), indicating that the results may be less affected by potential bias. At the same time, the MR-PRESSO method also obtained results consistent with the MR-Egger regression; that is, gene pleiotropy did not exist. Although in lung, squamous cell lung, breast, endometrial, and prostate cancers, the *P*-values of the MR-PRESSO analysis were less than 0.05, the results of the MR-PRESSO destruction test were supported by the absence of horizontal pleiotropy (*P* > 0.05). The MR-PRESSO distortion test here refers to whether there is a difference between the results after removing outlier SNP and the initial results (39). The detailed results are presented in Table 2.

**TABLE 2:** Horizontal pleiotropic test between dried fruit intake and eleven site-specific cancers.

Outcome	Horizontal pleiotropy test			MR-PRESSO	
	Intercept	*SE*	*P*-value	*P*-value	DT *P*-value
Oral cavity/pharyngeal cancer	0.041	0.0388	0.2976	0.65	
Lung cancer	−0.0095	0.0187	0.6153	0.005	0.686
Squamous cell lung cancer	−0.0268	0.0265	0.3199	0.046	0.757
Breast cancer	−0.0103	0.0101	0.3132	0.013	0.633
Ovarian cancer	0.0118	0.0116	0.3145	0.205	
Pancreatic cancer	0.1324	0.1593	0.4377	0.563	
Cervical cancer	−0.0001	0.0001	0.4277	0.461	
Lung adenocarcinoma	−0.0085	0.0244	0.7263	0.18	
Endometrial cancer	0.0159	0.0146	0.2847	0.038	0.202
Thyroid cancer	0.1141	0.2848	0.7051	0.305	
Prostate cancer	0.0148	0.0117	0.2134	<0.001	0.452
Bladder cancer	−0.00001	0.0001	0.8845	0.067	
Brain cancer	0.00004	0.0001	0.6709	0.157	

SE, standard error; DT, distortion test.

Leave-one-out analysis was used to analyze the results of the IVW method. After removing each SNP individually, the results were consistent with the IVW method in causal effect analysis, indicating that no single SNP affected the causal estimation results. The results are presented in Supplementary Figure 3. Cochran’s statistical test showed no statistically significant heterogeneity effect (*Q*-value > 0.05) of the SNP related to dried fruit intake between oral cavity/pharyngeal, ovarian, pancreatic, cervical, lung adenocarcinoma, thyroid, bladder, and brain cancers. Although the results for lung, squamous cell lung, breast, endometrial, and prostate cancers showed heterogeneity (*Q*-value < 0.05), the results of the multiple random effects model were consistent with MR estimates, indicating that there was a causal effect between dried fruit intake and lung, squamous cell lung, and breast cancers (*P* < 0.05); while no association with endometrial cancer, and prostate cancer (*P* > 0.05). The reliability of the results of this study can be explained further. The specific results are listed in Table 3.

**TABLE 3:** Heterogeneity test between dried fruit intake and eleven site-specific cancers.

Outcome and method	Cochran’s *Q* test			Multiplicative random effects		
	*Q*	*Q*_df	*Q*-value	Beta	*SE*	*P*-value
Oral cavity/pharyngeal cancer						
MR-Egger	28.012	32	0.6688			
IVW	29.1333	33	0.6602			
Lung cancer				−1.1091	0.3397	0.0011
MR-Egger	62.9339	37	0.0049			
IVW	63.3708	38	0.006			
Squamous cell lung cancer				−1.4697	0.488	0.0026
MR-Egger	54.303	37	0.033			
IVW	55.7951	38	0.0313			
Breast cancer				−0.7566	0.1857	4.62 × 10^–5^
MR-Egger	61.4347	39	0.0124			
IVW	63.0793	40	0.0114			
Ovarian cancer						
MR-Egger	45.9324	38	0.1765			
IVW	47.1886	39	0.1727			
Pancreatic cancer						
MR-Egger	5.6025	6	0.4692			
IVW	6.2934	7	0.5059			
Cervical cancer						
MR-Egger	36.2438	35	0.4104			
IVW	36.9106	36	0.4266			
Lung adenocarcinoma						
MR-Egger	45.6275	37	0.1562			
IVW	45.7808	38	0.1805			
Endometrial cancer				−0.0423	0.2671	0.8742
MR-Egger	56.3945	39	0.0353			
IVW	58.0962	40	0.032			
Thyroid cancer						
MR-Egger	7.2731	5	0.2011			
IVW	7.5067	6	0.2765			
Prostate cancer				0.1331	0.2148	0.5354
MR-Egger	128.1553	39	1.9813 × 10^–11^			
IVW	133.4129	40	5.5721 × 10^–12^			
Bladder cancer						
MR-Egger	48.6025	35	0.063			
IVW	48.6322	36	0.0778			
Brain cancer						
MR-Egger	45.3404	35	0.1132			
IVW	45.5783	36	0.1316			

Q, Cochran’s Q statistic; df, degrees of freedom; SE, standard error; IVW, inverse-variance-weighted.

### Multivariate Mendelian randomization analysis

**FIGURE 4:**
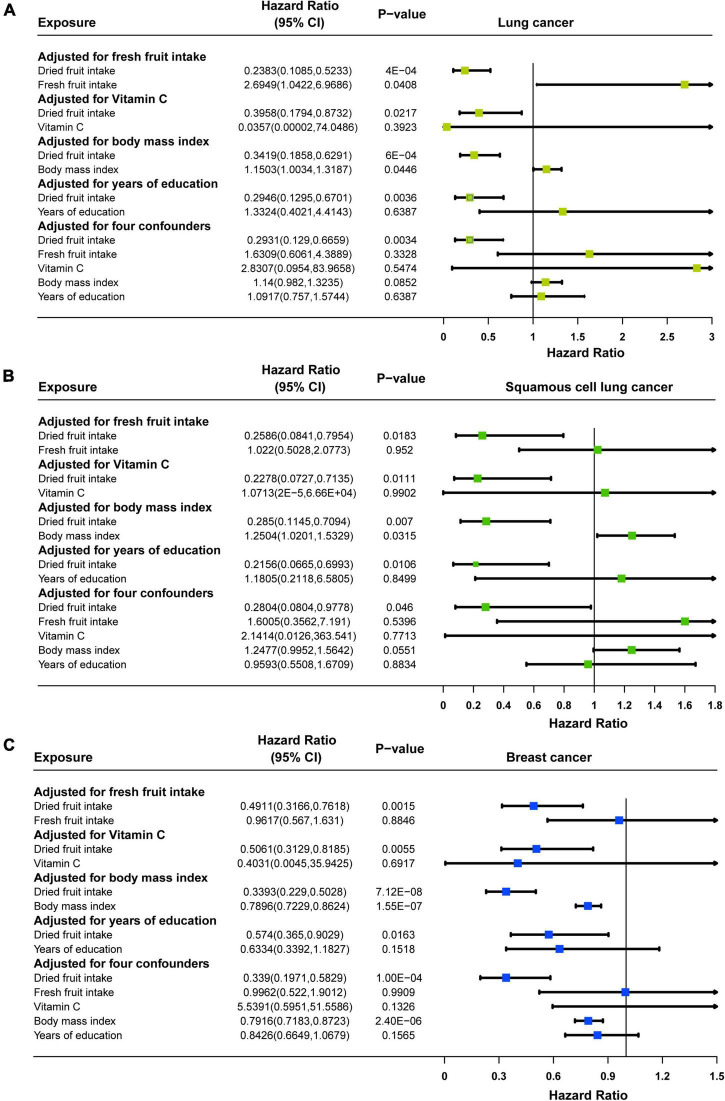
Forest plots of multivariable Mendelian randomization (MR) in **(A)** lung cancer, **(B)** squamous cell lung cancer, and **(C)** breast cancer. Adjusted for fresh fruit intake, vitamin C, body mass index, years of education or fresh fruit intake, vitamin C, body mass index, and years of education.

Multivariate MR analysis for each cancer found that: for lung cancer, after adjusting for fresh fruit intake (OR = 0.2383, 95% CI: 0.1085–0.5233, *P* = 0.0004), vitamin C (OR = 0.3958, 95% CI: 0.1794–0.8732, *P* = 0.0217), BMI (OR = 0.3419, 95% CI: 0.1858–0.6291, *P* = 0.0006), years of education (OR = 0.2946, 95% CI: 0.1295–0.6701, *P* = 0.0036), and all of these four (OR = 0.2931, 95% CI: 0.129–0.6659, *P* = 0.0034), dried fruit intake remained causally associated with lung cancer, and the effect size for causality was slightly enhanced in multivariate MR compared with univariate MR (Figure 4A); for squamous cell lung cancer, after adjusting for fresh fruit intake (OR = 0.2586, 95% CI: 0.0841–0.7954, *P* = 0.0183), vitamin C (OR = 0.2278, 95% CI: 0.0727–0.7135, *P* = 0.0111), BMI (OR = 0.285, 95% CI: 0.1145–0.7094, *P* = 0.007), years of education (OR = 0.2156, 95% CI: 0.0665–0.6993, *P* = 0.0106), and all of these four (OR = 0.2804, 95% CI: 0.0804–0.9778, *P* = 0.046), though the effect of causality was slightly attenuated in multivariate MR compared to univariate MR, dried fruit intake and squamous cell lung cancer remained causally associated (Figure 4B); for breast cancer, after adjusting for fresh fruit intake (OR = 0.4911, 95% CI: 0.3166–0.7618, *P* = 0.0015), vitamin C (OR = 0.5601, 95% CI: 0.3129–0.8185, *P* = 0.0055), BMI (OR = 0.3393, 95% CI: 0.229–0.5028, *P* = 7.12 × 10^–8^), years of education (OR = 0.574, 95% CI: 0.365–0.9029, *P* = 0.0163), and all of these four (OR = 0.339, 95% CI: 0.1971–0.5829, *P* = 0.0001), dried fruit intake remained causally associated with breast cancer, and the effect size for causality was slightly enhanced in multivariate MR compared to univariate MR (Figure 4C). However, for oral cavity/pharyngeal, ovarian, cervical, pancreatic, lung adenocarcinoma, endometrial, thyroid, prostate, bladder, and brain cancers, after adjustment for multivariate MR, the causal relationship between dried fruit intake and outcome was not statistically significant (Supplementary Figure 4).

### Validation

**FIGURE 5:**
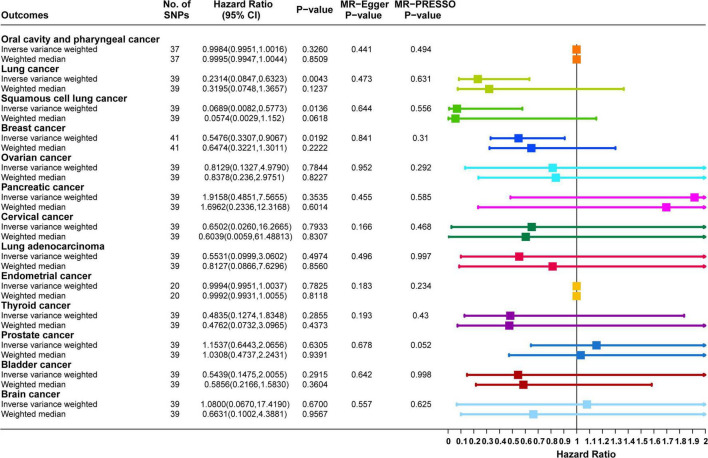
Forest plot of two-sample Mendelian randomization (MR) estimation of the association between dried fruit intake and cancer risk in validation datasets. No. of SNPs, number of single nucleotide polymorphisms; CI, confidence interval.

In the validation cohort, the results of the IVW method supported a causal relationship between dried fruit intake and lung, squamous cell lung, and breast cancers. The higher the dried fruit intake, the lower the cancer incidence risk. For every increase in dried fruit intake by one standard deviation, lung cancer risk was reduced by 76.86% (OR = 0.2314, 95% CI: 0.0847–0.6323, *P* = 0.0043); squamous cell lung cancer risk was reduced by 93.11% (OR = 0.0689, 95% CI: 0.0082–0.5773, *P* = 0.0136); and breast cancer risk was reduced by 45.24% (OR = 0.5476, 95% CI: 0.3307–0.9067, *P* = 0.0192); however, for oral cavity/pharyngeal, ovarian, cervical, pancreatic, lung adenocarcinoma, endometrial, thyroid, prostate, bladder, and brain cancers, neither the IVW nor WM methods showed statistical significance. The details of the results are shown in Figure 5. The MR-Egger regression analysis and MR-PRESSO method excluded the effect of horizontal multiplicity on causality to some extent (Figure 5). Cochran’s statistical test found no significant statistical effect of heterogeneity on causality estimates, ensuring the robustness of the results (Supplementary Table 4). For endometrial cancer (corrected *P* value = 0.4793) and oral cavity/pharyngeal cancer (corrected *P* value = 0.9565), the analysis results of “*MRlap*” showed that after adjusting for the impact of sample overlap, the causal effects between dried fruit intake and these two cancers were consistent to the results of two-sample MR.

## Discussion

This study used a two-sample MR method to explore the relationship between dried fruit intake and 11 site-specific cancers in the European population. The results showed a causal relationship between dried fruit intake and oral cavity/pharyngeal, lung, squamous cell lung, breast, ovarian, pancreatic, and cervical cancers. However, no causal relationship was observed with lung adenocarcinoma, endometrial, thyroid, prostate, bladder, and brain cancers. In addition, the causal relationships between dried fruit intake and lung, squamous cell lung, and breast cancers were validated using the validation datasets. To our knowledge, this is the first study to focus on the causal relationship between dried fruit intake and cancer by using MR analysis.

Dried fruits are favored because of their sweet taste, stability, and ease of preservation. By drying fresh fruit, it retains as many nutrients as possible from the original food, which is also why it is a good source of fiber and some trace elements (40, 41). In addition, dried fruits are rich in a wide range of bioactive components and phytochemicals. Because these compounds are not necessary to maintain life, they are not designated as traditional nutrients; however, the benefits of plant compounds may exceed human cognition. They can affect the occurrence and development of many chronic diseases by affecting metabolic pathways and cellular reactions and play a role in promoting health and longevity (42). However, people have preferred other processed fruits in recent years, such as pickled and fermented fruits; the consumption of dried fruits is even lower than that of canned fruits (43, 44). Therefore, this study focused on dried fruit as an exposure factor to explore the causal relationship between dried fruit intake and cancer and provide a new entry point for cancer prevention.

A cohort study of 61 lung cancer patients in the United States showed that there was a statistically significant protective association between dried fruit intake and lung cancer (dried fruit intake less than three times per week, RR = 1.0; greater than or equal to three times per week, RR = 0.89) (45); After adjusting for age and sex, dried fruit intake was also a protective association with pancreatic cancer (dried fruit intake was less than one times per month, RR = 1; greater than or equal to three times per week, RR = 0.35) (46). The results of two prospective cohorts and one case-control study showed a protective trend for prostate cancer; however, only one study was statistically significant (47–49). These studies are consistent with the conclusion of this study. The MR method has its unique advantages because genes have been determined at human birth, SNP as an IV will not be affected by various confounding factors, and the reasonable temporal sequence in causal inference guaranteed the reliability of the conclusion.

As mentioned, causal estimation is effective when the three assumptions in the MR model are satisfied. First, 43 significantly correlated and independent SNPs loci were selected that were closely related to dried fruit intake. At the same time, the F-statistic for each SNP was greater than 10, indicating that the selected SNPs were robust IVs. Second, the data in this study were from the European population, which avoided the bias caused by different populations to a certain extent. Third, to evaluate the bias caused by pleiotropy in MR, we used the MR-Egger regression method and found that the intercept was close to 0 (*P* > 0.05), indicating that unknown factors caused no pleiotropy. Additionally, no pleiotropy was observed in the results of the MR-PRESSO method. Third, the heterogeneity test results support the lack of heterogeneity in our results. Therefore, the selected IVs and study results were reliable.

It is widely believed that the intake of fresh fruit can reduce the risk of cancer. Some studies have shown that fresh fruit intake had a significant protective effect on the oral cavity/pharyngeal (50), lung (51), and breast cancers (52, 53), but no significant effect on ovarian (54), pancreatic (51), endometrial (51), thyroid (55), prostate (56), bladder (57), and cervical cancers (58). In this regard, we performed a two-sample MR analysis between fresh fruit intake as exposure and 11 site-specific cancers as outcome. The evidence from IVW analysis showed that each increase of fresh fruit intake by one standard development was statistically significantly associated with 35.06% decrease of breast cancer incidence risk (*P* = 0.0365); however, there was no significant effect on oral cavity/pharyngeal (*P* = 0.0533), lung (*P* = 0.8809), squamous cell lung (*P* = 0.2163), ovarian (*P* = 0.0969), pancreatic (*P* = 0.0734), lung adenocarcinoma (*P* = 0.5806), endometrial (*P* = 0.5982), thyroid (*P* = 0.7896), prostate (*P* = 0.1772), bladder (*P* = 0.41), cervical (*P* = 0.4315), and brain cancers (*P* = 0.0703), this is basically consistent with the research conclusion of other researchers. The further details of the results are shown in Supplementary Figure 5 and Supplementary Table 5.

Our study suggests that intake of dried fruit has potential preventive value against some site-specific cancers. Interventions in dried fruit intake may help reduce the risk of some cancers. Active health education based on dried fruit intake and reasonable adjustment of the diet ratio may help improve human quality of life. Besides, the protective effect of dried fruit intake on site-specific cancers is no less than that of fresh fruit intake. Fresh fruit consumption is usually affected by seasonal factors (59), so intake of dried fruit can be another good choice.

The impact of dried fruit intake on cancer, the relevant research is not perfect at present, but some studies mentioned that, numerous beneficial phytochemicals are conserved even after processing of fruits to be dried fruits, therefore, intake of dried fruits can help prevent cancer (60). From another point of view, the potential mechanisms of the effects of both fresh and dried fruit on cancer need to be further explored. Research on the potential mechanism behind the protective effect of dried fruits on some cancers may support the pharmacological development of cancer prevention and treatment.

However, our study has some limitations. First, the participants in this study were all of European ancestry; extrapolation of the conclusion to other populations has certain limitations. Nevertheless, we have tried our best to ensure that the research results are not disturbed by other populations and increase the possibility of extrapolation. Second, we analyzed only 11 eleven site-specific cancers, which is the site-specific cancer data that we can obtain to the greatest extent. If possible, we will continue exploring other site-specific cancers to understand the relationship between dried fruit intake and cancers fully. Third, the MR method can only analyze the causal relationship but cannot explain the mechanism behind the protective effect of dried fruit intake on some cancers. Further experimental studies are needed to explore the mechanism of the impact of dried fruit intake on the risk of cancer.

## Data Availability Statement

The original contributions presented in this study are included in the article/Supplementary Material, further inquiries can be directed to the corresponding authors.

## Author contributions

YW and GC conceptualized and designed the study and had full access to all the data in the study and had responsibility for the integrity of the data, the accuracy of the analyses, and the final decision to submit the manuscript for publication. CJ, RL, TD, ZL, HL, YaY, QS, JiW, YiY, and JuW collected the data and performed the analysis. CJ drafted the initial version of the manuscript. All authors contributed to results interpretation, critically reviewed many revisions of the manuscript, and contributed to important intellectual content.

## Conflict of Interest

The authors declare that the research was conducted in the absence of any commercial or financial relationships that could be construed as a potential conflict of interest.

## Publisher’s Note

All claims expressed in this article are solely those of the authors and do not necessarily represent those of their affiliated organizations, or those of the publisher, the editors and the reviewers. Any product that may be evaluated in this article, or claim that may be made by its manufacturer, is not guaranteed or endorsed by the publisher.

## Footnotes

1
http://www.phenoscanner.medschl.cam.ac.uk/
