# Supplementary material for: Association between dried fruit intake and pan-cancers incidence risk: A two-sample Mendelian randomization study
Source: Front Nutr. 2022 Jul 18;9:899137. doi: 10.3389/fnut.2022.899137 (PMC9339715; doi:10.3389/fnut.2022.899137)
Supplement: Supplementary file 1 [file Data_Sheet_1.docx]

# Supplementary Materials


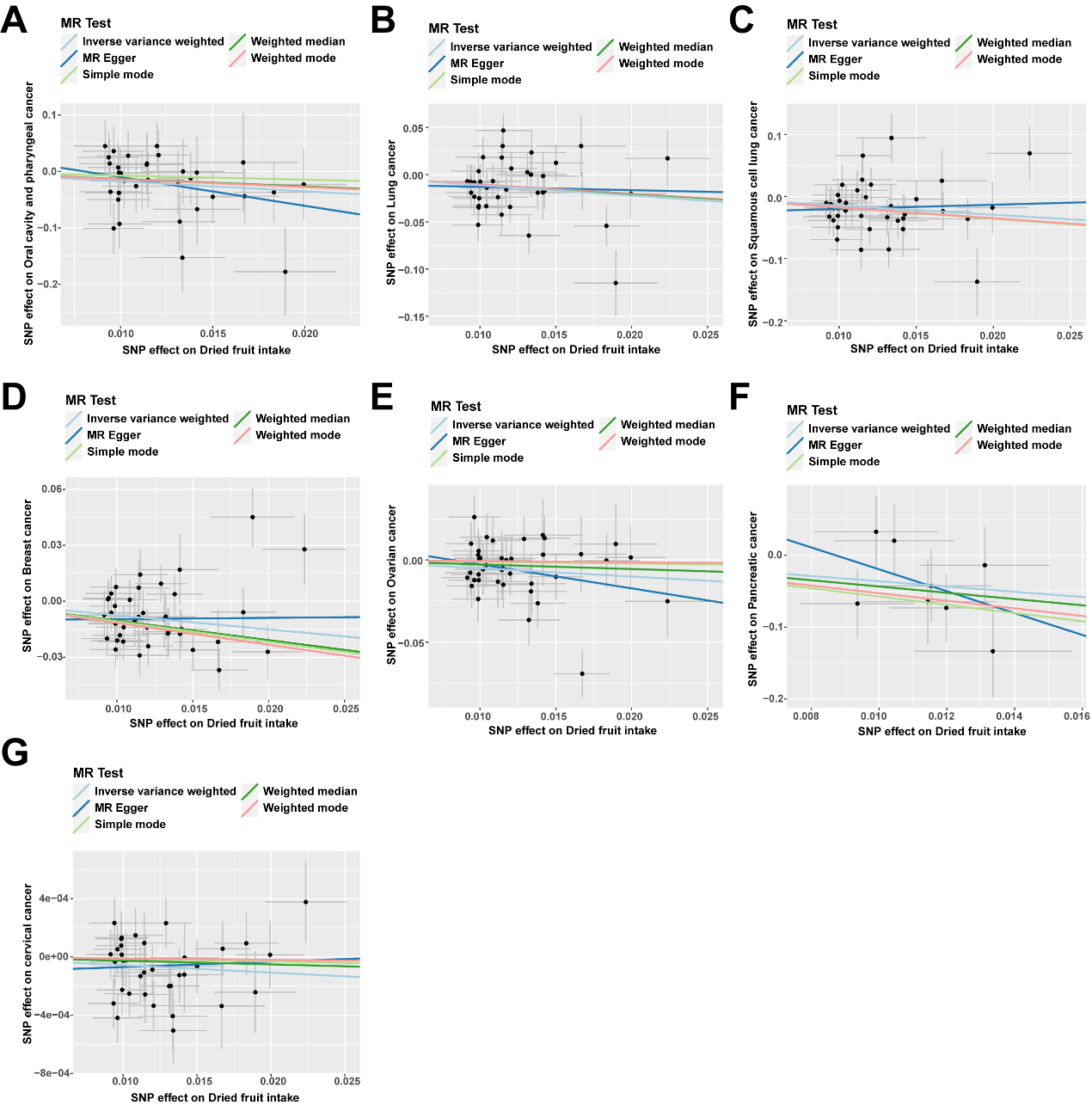


**Supplementary Figure 1-1 |** Scatter plot of SNPs associated with dried fruit intake and risk on **(A)** oral cavity/pharyngeal cancer, **(B)** lung cancer, **(C)** squamous cell lung cancer, **(D)** breast cancer, **(E)** ovarian cancer, **(F)** pancreatic cancer, and **(G)** cervical cancer incidence.


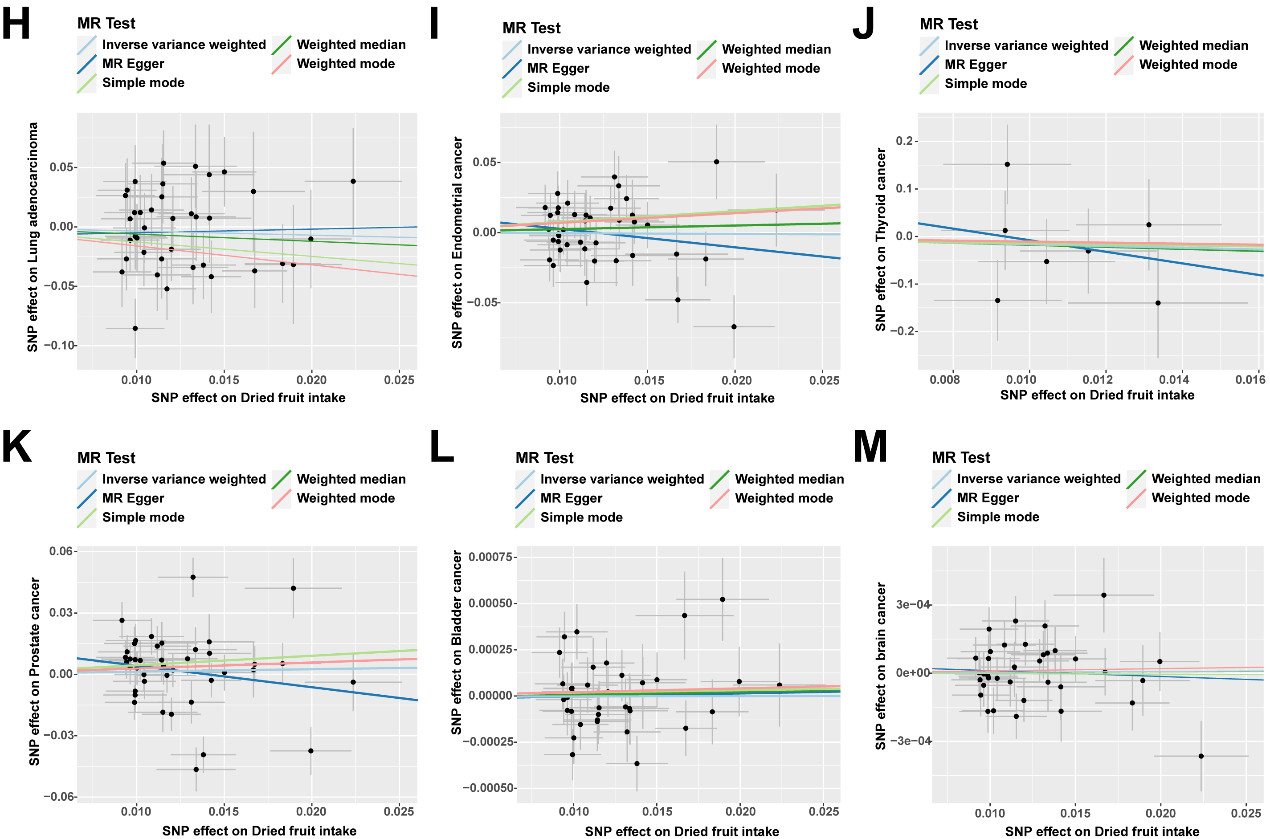


**Supplementary Figure 1-2 |** Scatter plot of SNPs associated with dried fruit intake and risk on **(H)** lung adenocarcinoma, **(I)** endometrial cancer, **(J)** thyroid cancer, **(K)** prostate cancer, **(L)** bladder cancer, and **(M)** brain cancer incidence.


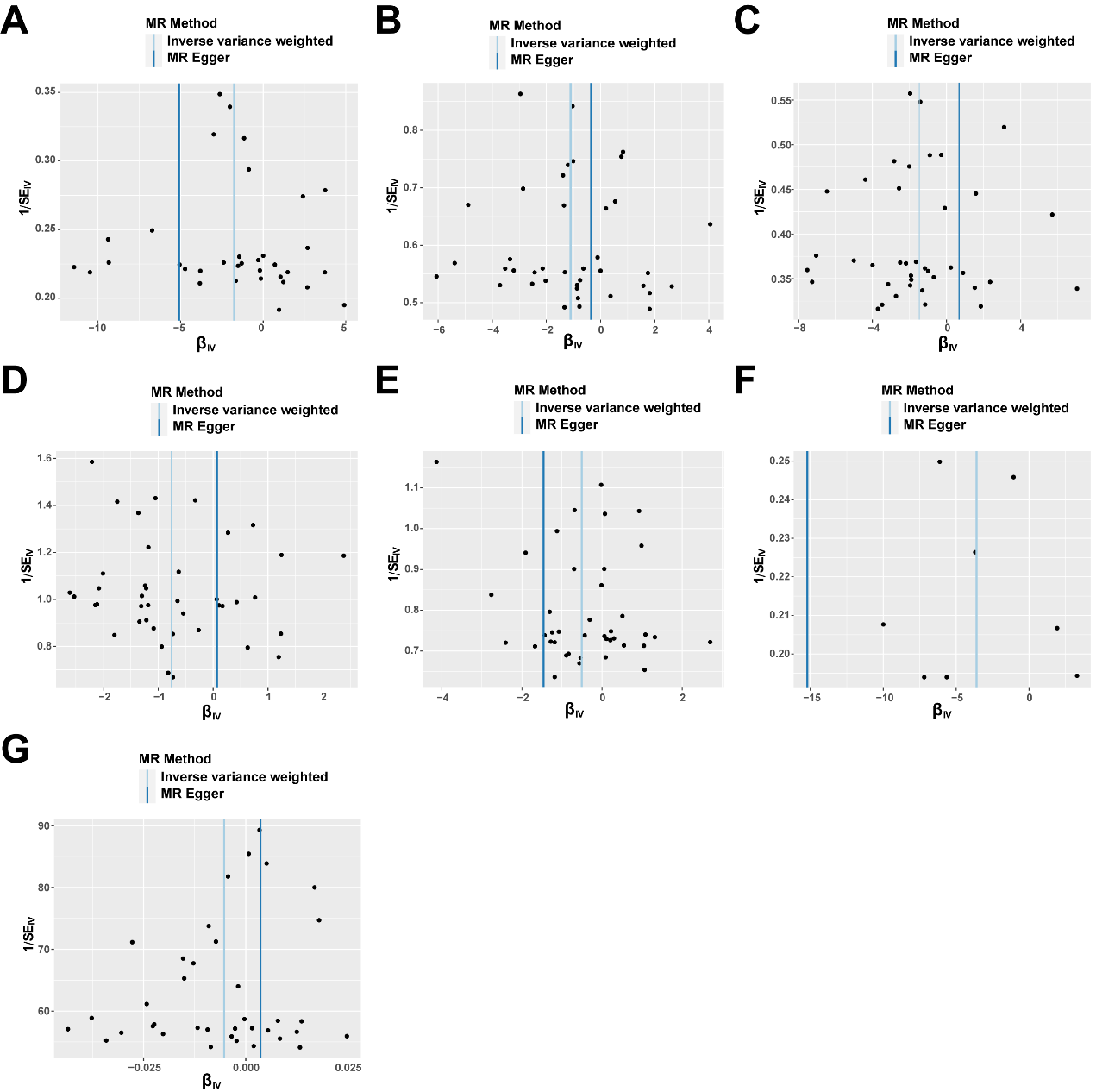


**Supplementary Figure 2-1 |** Funnel plot of the relationship between the causal effect of dried fruit intake on site-specific cancer and the reciprocal of the standard deviation of the causal estimation using a single SNP as an instrument. **(A)** oral cavity/pharyngeal cancer, **(B)** lung cancer, **(C)** squamous cell lung cancer, **(D)** breast cancer, **(E)** ovarian cancer, **(F)** pancreatic cancer, and **(G)** cervical cancer.


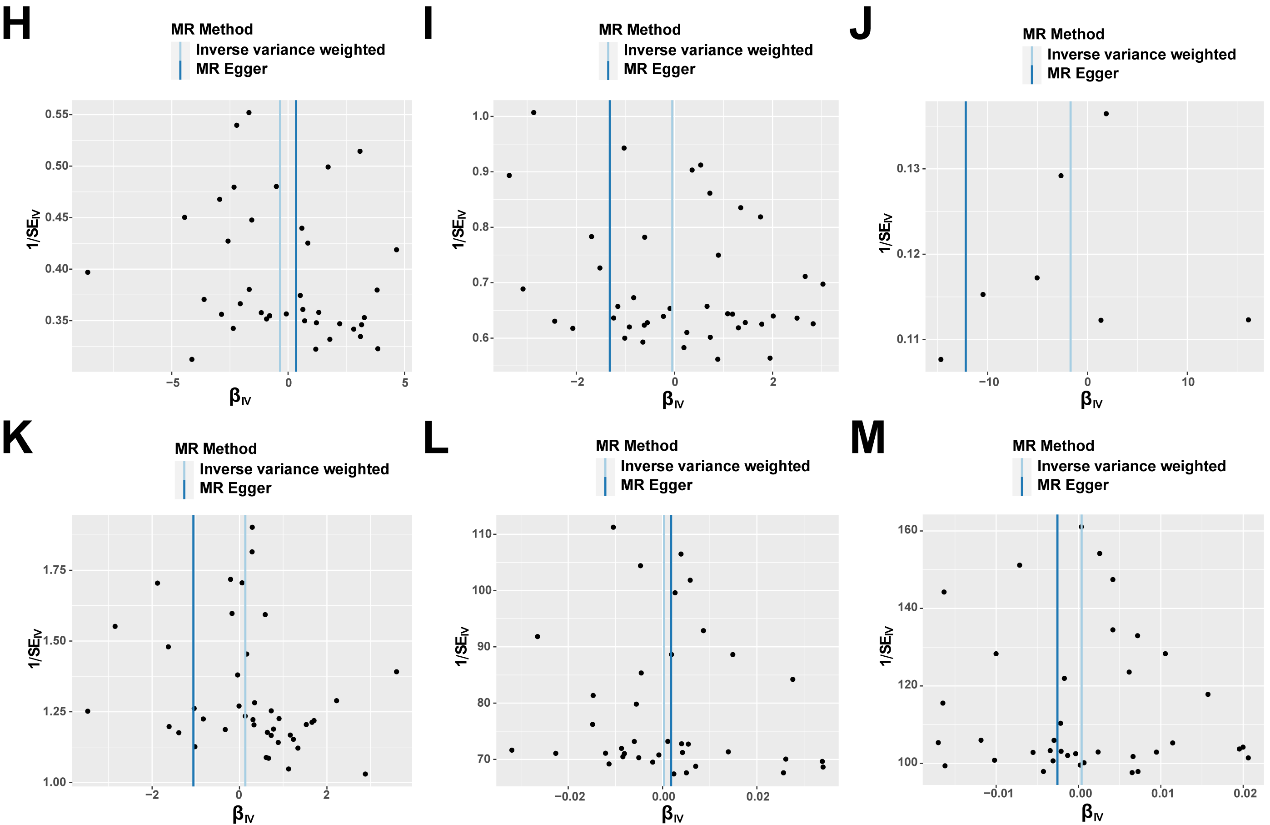


**Supplementary Figure 2-2 |** Funnel plot of the relationship between the causal effect of dried fruit intake on site-specific cancer and the reciprocal of the standard deviation of the causal estimation using a single SNP as an instrument. **(H)** lung adenocarcinoma, **(I)** endometrial cancer, **(J)** thyroid cancer, **(K)** prostate cancer, **(L)** bladder cancer, and **(M)** brain cancer.


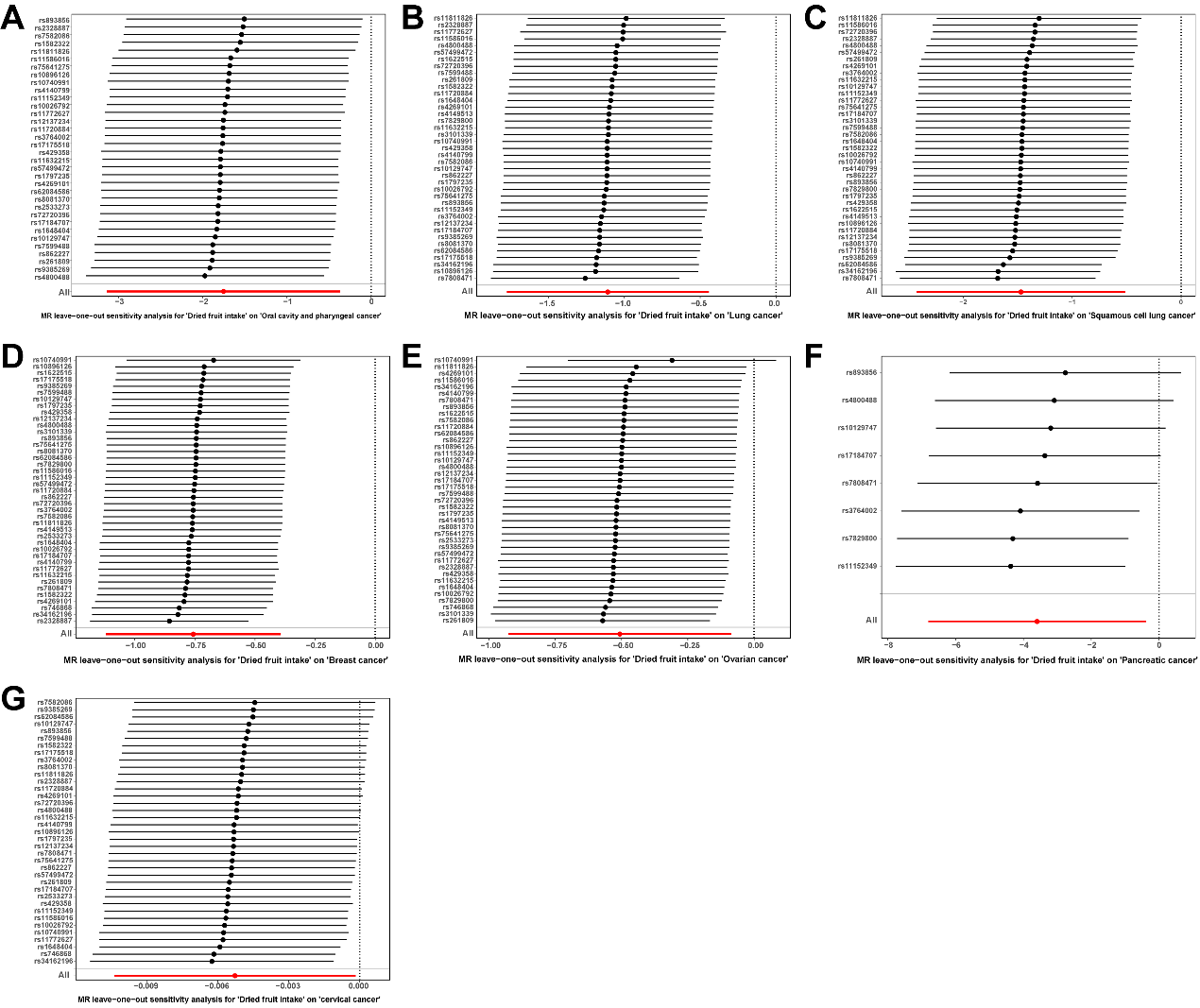


**Supplementary Figure 3-1 |** Leave-one-out analysis result of SNPs associated with dried fruit intake and risk on **(A)** oral cavity/pharyngeal cancer, **(B)** lung cancer, **(C)** squamous cell lung cancer, **(D)** breast cancer, **(E)** ovarian cancer, **(F)** pancreatic cancer, and **(G)** cervical cancer incidence.


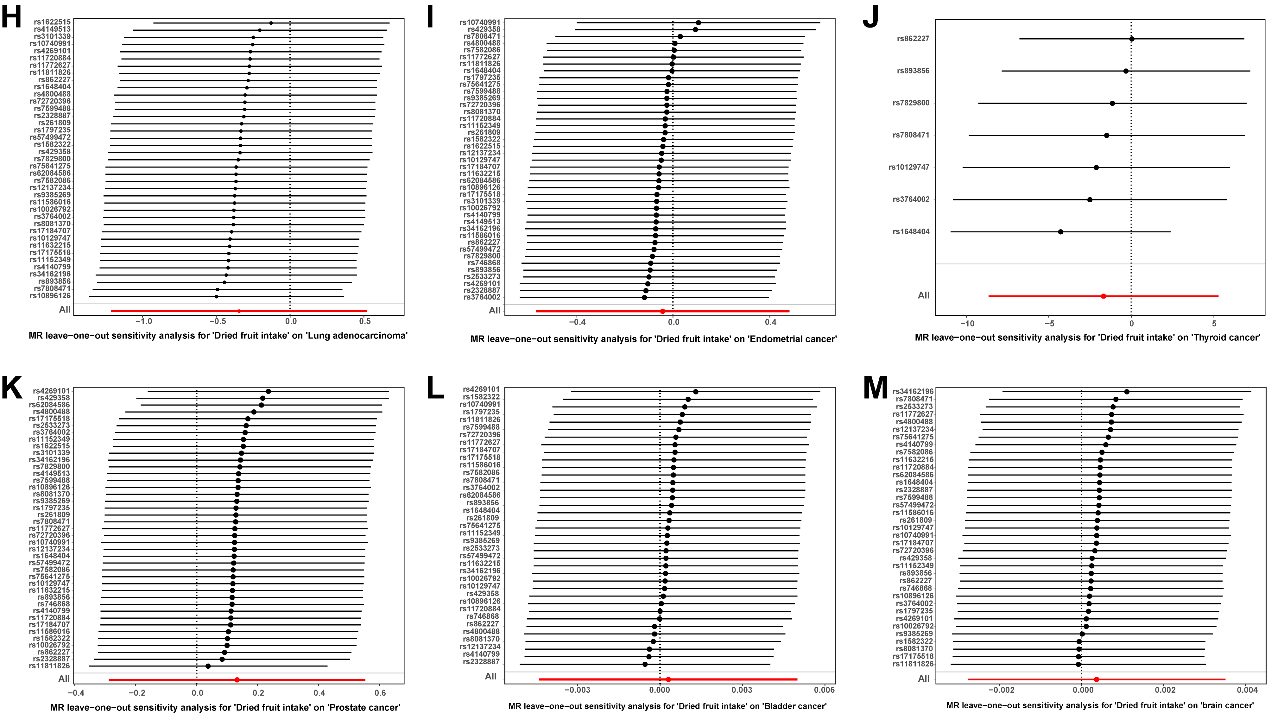


**Supplementary Figure 3-2 |** Leave-one-out analysis result of SNPs associated with dried fruit intake and risk on **(H)** lung adenocarcinoma, **(I)** endometrial cancer, **(J)** thyroid cancer, **(K)** prostate cancer, **(L)** bladder cancer, and **(M)** brain cancer incidence.


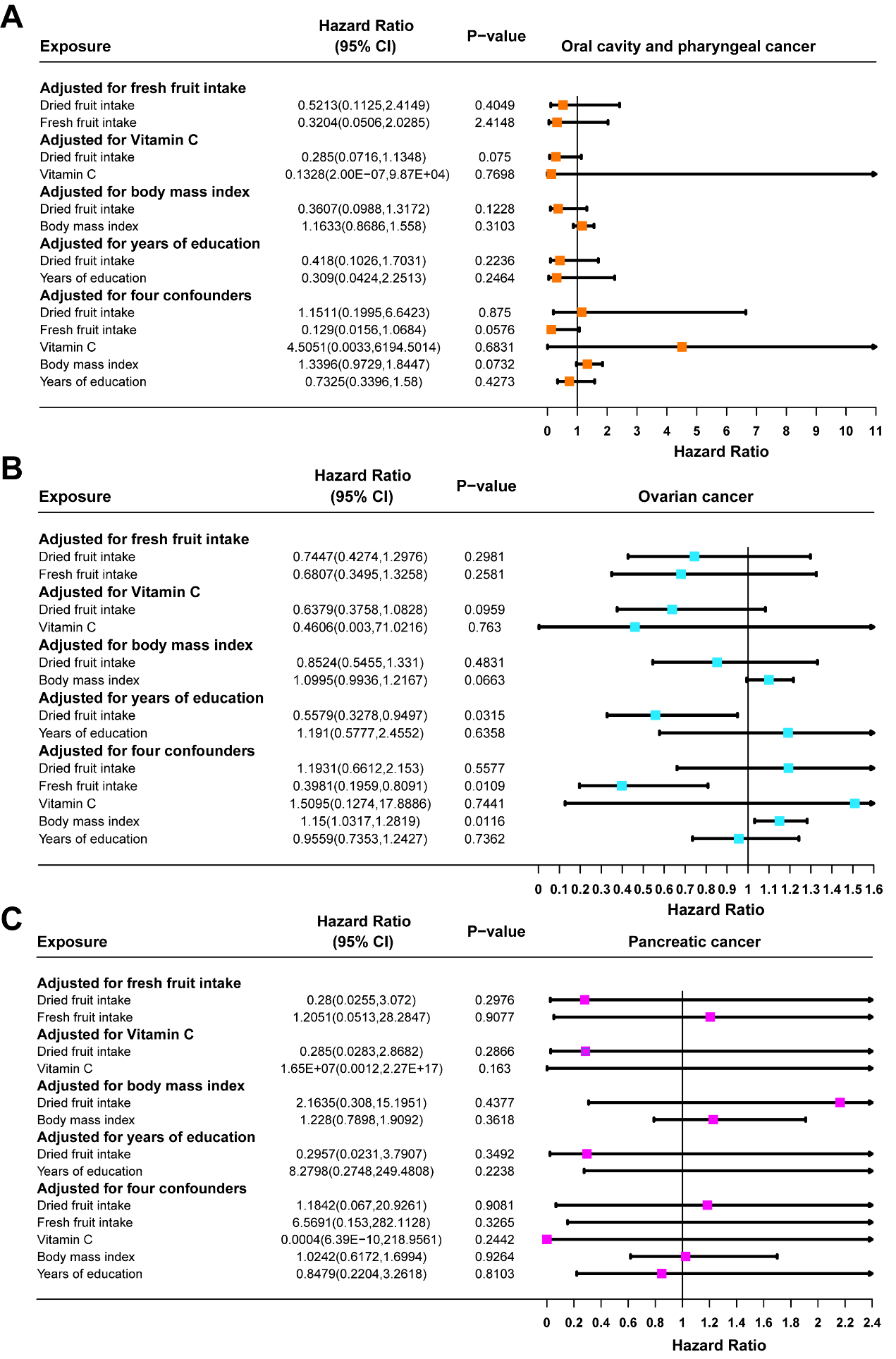


**Supplementary Figure 4-1 |** Forest plots of multivariable MR in **(A)** oral cavity/pharyngeal cancer, **(B)** ovarian cancer, and **(C)** pancreatic cancer. Adjusted for fresh fruit intake, vitamin C, body mass index, years of education or fresh fruit intake, vitamin C, body mass index, and years of education.


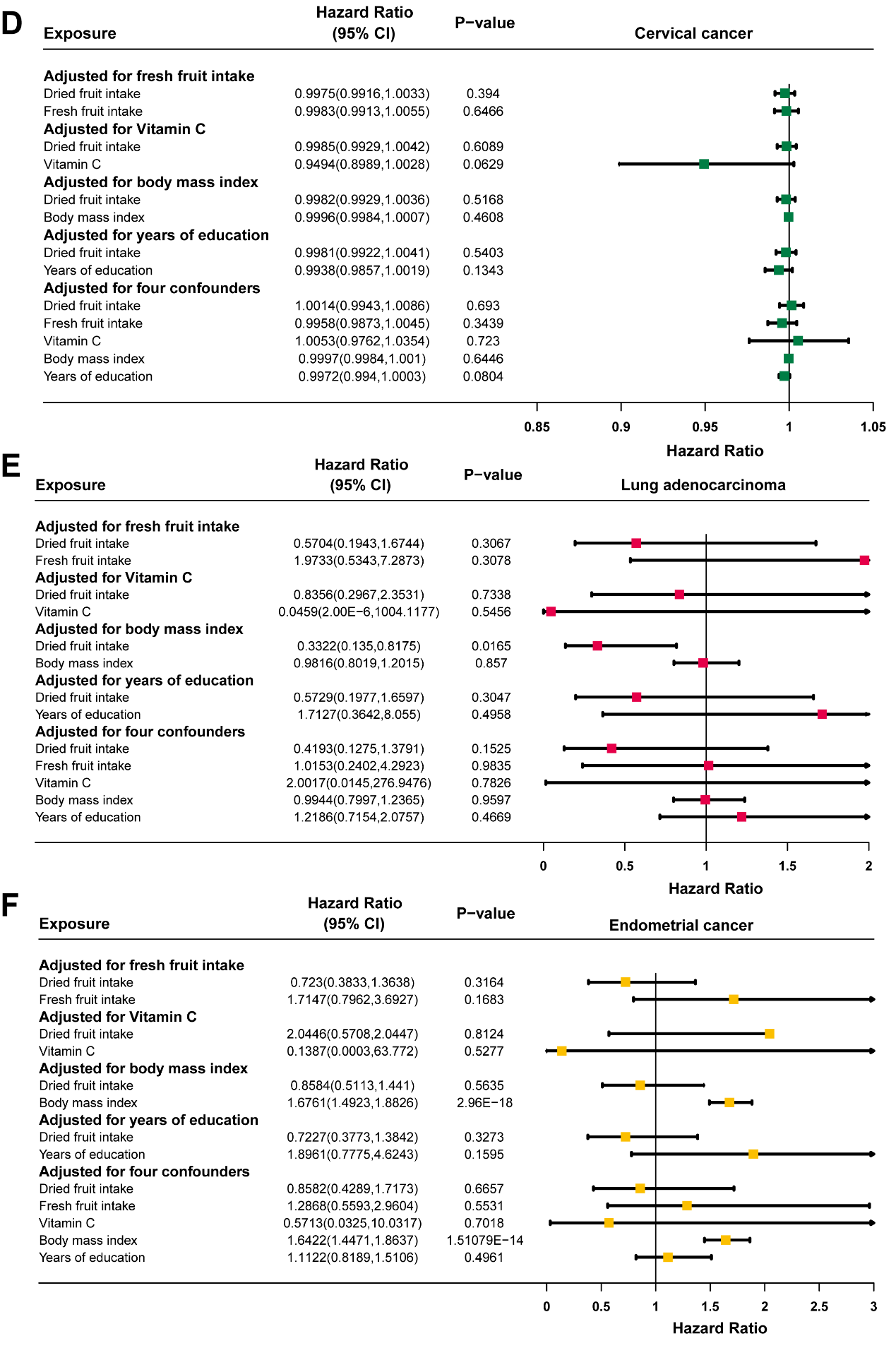


**Supplementary Figure 4-2 |** Forest plots of multivariable MR in **(D)** cervical cancer, **(E)** lung adenocarcinoma, and **(F)** endometrial cancer. Adjusted for fresh fruit intake, vitamin C, body mass index, years of education or fresh fruit intake, vitamin C, body mass index, and years of education.


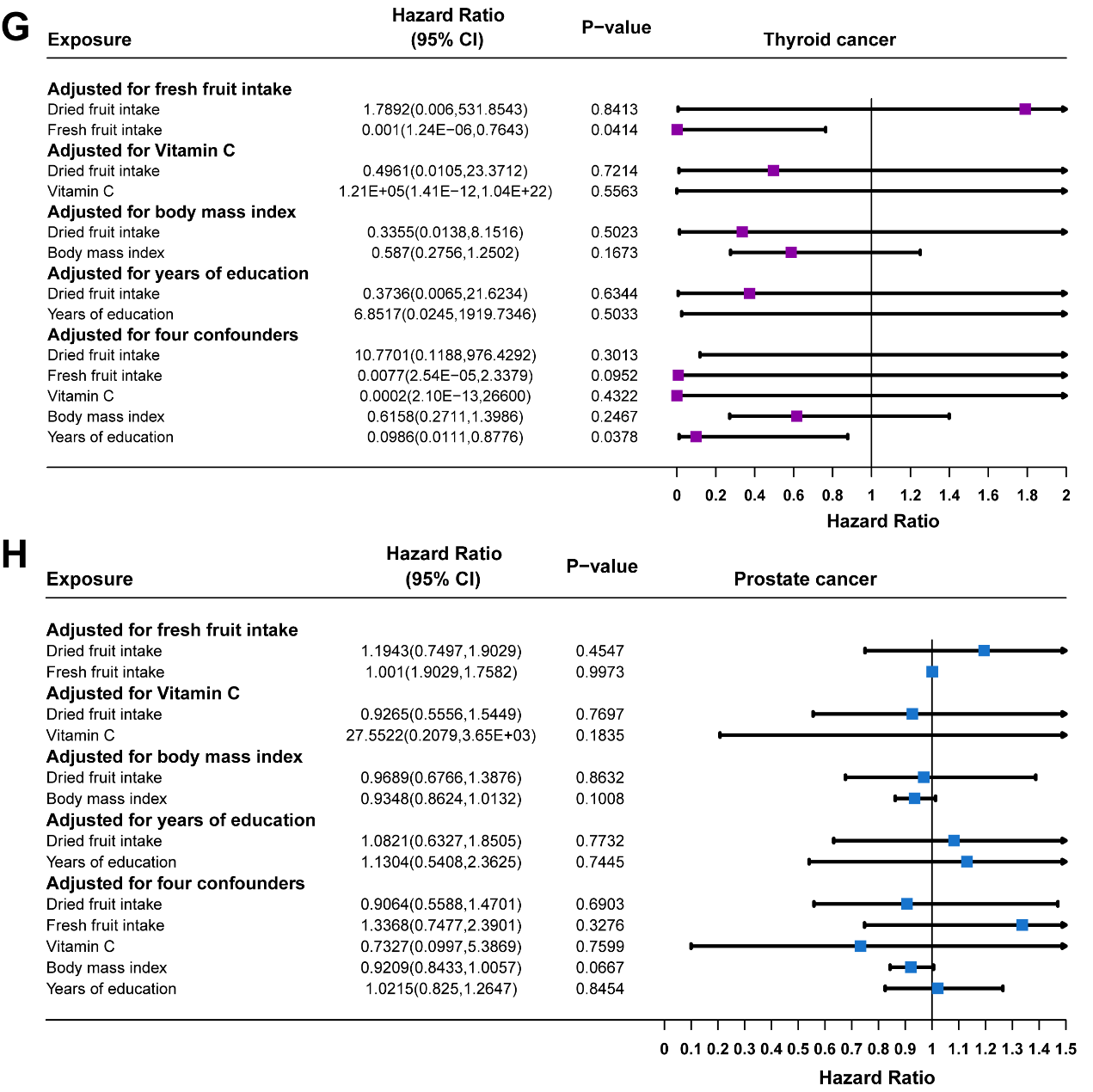


**Supplementary Figure 4-3 |** Forest plots of multivariable MR in **(G)** thyroid cancer **(H)** and prostate cancer. Adjusted for fresh fruit intake, vitamin C, body mass index, years of education or fresh fruit intake, vitamin C, body mass index, and years of education.


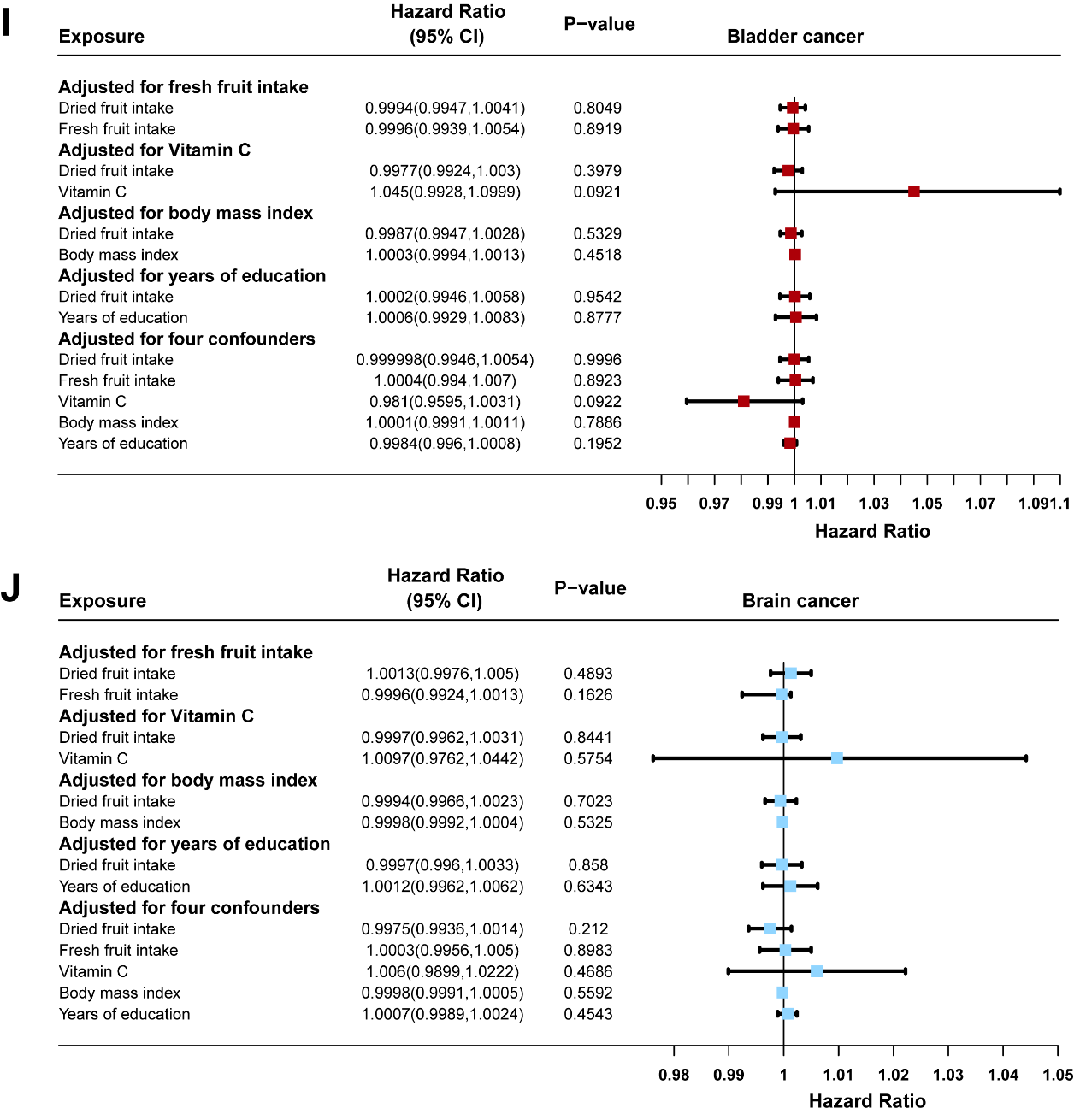


**Supplementary Figure 4-4 |** Forest plots of multivariable MR in **(I)** bladder cancer and **(J)** brain cancer. Adjusted for fresh fruit intake, vitamin C, body mass index, years of education or fresh fruit intake, vitamin C, body mass index, and years of education.

**
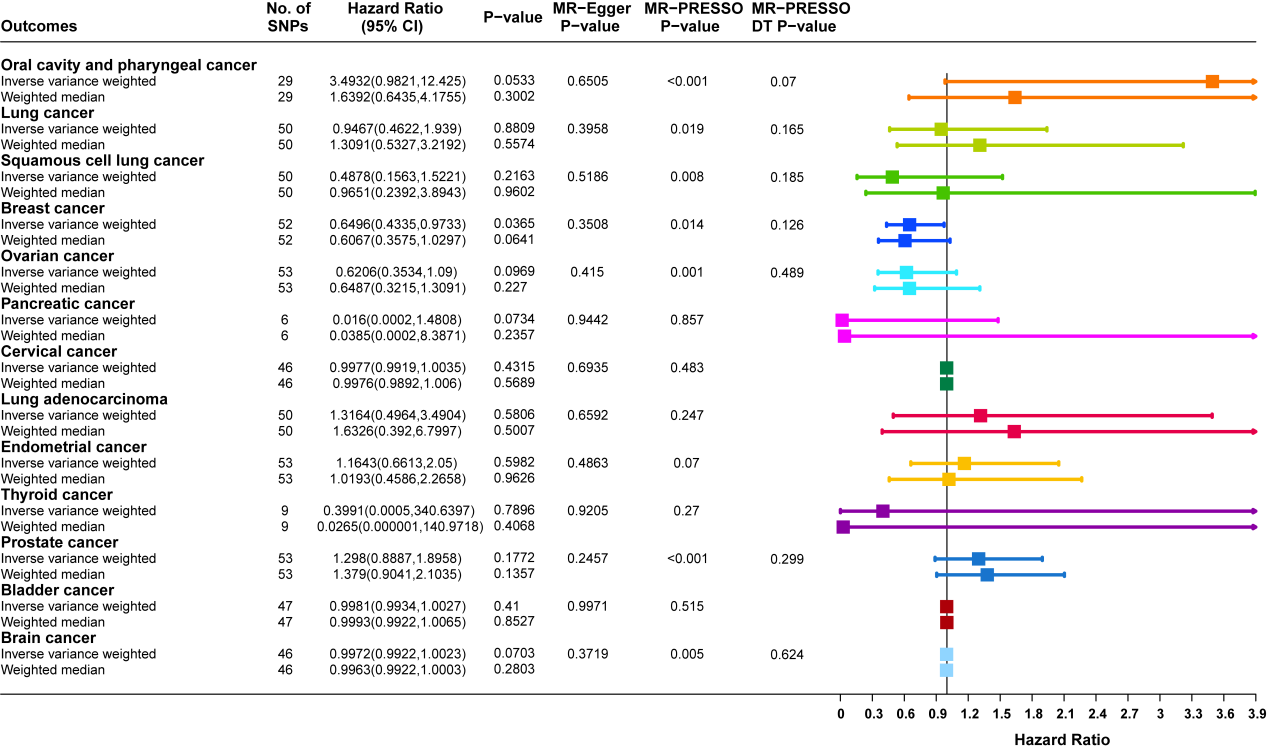
**

**Supplementary** **Figure 5 |** Forest plot of two-sample Mendelian randomization (MR) estimation of the association between fresh fruit intake and cancer risk. No. of SNPs: Number of single nucleotide polymorphisms; CI: confidence interval.

**Supplementary Table 1** **|** The sources for all statistical summary datasets used in this study

| **Usage purposes** | **Phenotype** | **Source** | **GWAS ID** | **Population** |
| --- | --- | --- | --- | --- |
| Univariate two-sample MR analysis & MR in Validation datasets & Multivariate MR analysis | Dried fruit intake | <https://gwas.mrcieu.ac.uk/> | ukb-b-16576 | European |
| Univariate two-sample MR analysis | Fresh fruit intake | <https://gwas.mrcieu.ac.uk/> | ukb-b-3881 | European |
| Univariate two-sample MR analysis & Multivariate MR analysis | Oral cavity/pharyngeal cancer | <https://gwas.mrcieu.ac.uk/> | ieu-b-90 | European |
|  | Lung cancer | <https://gwas.mrcieu.ac.uk/> | ieu-a-966 | European |
|  | Squamous cell lung cancer | <https://gwas.mrcieu.ac.uk/> | ieu-a-967 | European |
|  | Breast cancer | <https://gwas.mrcieu.ac.uk/> | ieu-a-1168 | European |
|  | Ovarian cancer | <https://gwas.mrcieu.ac.uk/> | ieu-a-1120 | European |
|  | Pancreatic cancer | <https://gwas.mrcieu.ac.uk/> | ieu-a-822 | European |
|  | Lung adenocarcinoma | <https://gwas.mrcieu.ac.uk/> | ieu-a-965 | European |
|  | Endometrial cancer | <https://gwas.mrcieu.ac.uk/> | ebi-a-GCST006464 | European |
|  | Thyroid cancer | <https://gwas.mrcieu.ac.uk/> | ieu-a-1082 | European |
|  | Prostate cancer | <https://gwas.mrcieu.ac.uk/> | ebi-a-GCST006085 | European |
|  | Bladder cancer | <https://gwas.mrcieu.ac.uk/> | ieu-b-4874 | European |
|  | Brain cancer | <https://gwas.mrcieu.ac.uk/> | ieu-b-4875 | European |
|  | Cervical cancer | <https://gwas.mrcieu.ac.uk/> | ieu-b-4876 | European |
| Multivariate MR analysis | BMI | <https://gwas.mrcieu.ac.uk/> | ukb-a-248 | European |
|  | Years of education | <https://gwas.mrcieu.ac.uk/> | ieu-b-4835 | European |
|  | Fresh fruit intake | <https://gwas.mrcieu.ac.uk/> | ukb-b-3881 | European |
|  | Vitamin C | <https://gwas.mrcieu.ac.uk/> | ukb-b-15175 | European |
| MR in Validation datasets | Oral cavity/pharyngeal cancer | <https://gwas.mrcieu.ac.uk/> | ieu-b-4962 | European |
|  | Endometrial cancer | <https://gwas.mrcieu.ac.uk/> | ukb-b-13545 | European |
|  | Squamous cell lung cancer | <https://r6.finngen.fi/> | NA | European |
|  | Breast cancer | <https://r6.finngen.fi/> | NA | European |
|  | Ovarian cancer | <https://r6.finngen.fi/> | NA | European |
|  | Pancreatic cancer | <https://r6.finngen.fi/> | NA | European |
|  | Lung adenocarcinoma | <https://r6.finngen.fi/> | NA | European |
|  | Lung cancer | <https://r6.finngen.fi/> | NA | European |
|  | Thyroid cancer | <https://r6.finngen.fi/> | NA | European |
|  | Prostate cancer | <https://r6.finngen.fi/> | NA | European |
|  | Bladder cancer | <https://r6.finngen.fi/> | NA | European |
|  | Brain cancer | <https://r6.finngen.fi/> | NA | European |
|  | Cervical cancer | <https://r6.finngen.fi/> | NA | European |

**Supplementary Table 2** **|** Details of phenotypes related to 43 instrumental variables found on the Phenoscanner website

| **SNP** | **Trait & Source** | **Gene** | **Position (hg19):** |
| --- | --- | --- | --- |
| rs261809 | NA |  |  |
| rs11586016 | Comparative height size at age 10 (UKBB); Qualifications: college or university degree (UKBB); Years of educational attainment (PMID = 27225129); Height (UKBB); Qualifications: none (UKBB); Sitting height (UKBB); Qualifications: A levels or as levels or equivalent (UKBB) | PTPRF | chr1:44031793 |
| rs12137234 | NA |  |  |
| rs72720396 | Morning or evening person (UKBB); Alcohol usually taken with meals (UKBB); Average weekly beer plus cider intake (UKBB); Chronotype (PMID = 27494321); Morning vs evening chronotype (PMID = 27494321) | BARHL2 | chr1:91191582 |
| rs11811826 | Qualifications: college or university degree (UKBB); Years of educational attainment (PMID = 27225129); Qualifications: other professional qualifications (UKBB) | LRRN2 | chr1:204603861 |
| rs3101339 | Body mass index (UKBB); Weight (UKBB); Leg fat mass right (UKBB); Hip circumference (UKBB); Arm fat mass left (UKBB); Leg fat percentage left (UKBB); Arm fat mass right (UKBB); Trunk fat mass (UKBB); Qualifications: college or university degree (UKBB); Body fat percentage (UKBB); Waist circumference (UKBB) | NEGR1 | chr1:72748669 |
| rs75641275 | Body mass index (UKBB); Weight (UKBB); Years of educational attainment (PMID = 27225129); Trunk fat percentage (UKBB); Leg fat percentage left (UKBB); Trunk fat mass (UKBB); Body fat percentage (UKBB); Whole body fat mass (UKBB); Waist circumference (UKBB) | DPYD | chr1:98327133 |
| rs7582086 | Height (UKBB) | RP11-444A22.1 | chr2:60231826 |
| rs7599488 | Mean corpuscular volume (PMID = 27863252); Red blood cell count (PMID = 27863252); Time spent watching television (UKBB); Red cell distribution width (PMID = 27863252); Years of educational attainment (PMID = 27225129); Qualifications: O levels or GCSEs or equivalent (UKBB); Height (UKBB) | BCL11A | chr2:60718347 |
| rs4149513 | Qualifications: college or university degree (UKBB); Years of educational attainment (PMID = 27225129); Body fat percentage (UKBB); Leg fat percentage right (UKBB); Leg fat mass right (UKBB); Leg fat percentage left (UKBB) | CHST10 | chr2:101022726 |
| rs17184707 | NA |  |  |
| rs4269101 | Arm fat percentage right (UKBB); Body fat percentage (UKBB); Trunk fat percentage (UKBB); Arm fat percentage left (UKBB) | SATB1-AS1 | chr3:18763543 |
| rs11720884 | Leg fat percentage left (UKBB); Leg fat percentage right (UKBB); Leg fat mass right (UKBB); Leg fat mass left (UKBB) | RP11-944L7.4 | chr3:43941406 |
| rs57499472 | NA |  |  |
| rs10026792 | Overall health rating (UKBB); Qualifications: college or university degree (UKBB) | ADD1 | chr4:2862190 |
| rs1648404 | NA |  |  |
| rs746868 | Height (UKBB); Intestinal malabsorption (UKBB); Self-reported malabsorption or coeliac disease (UKBB); Primary sclerosing cholangitis (PMID = 27992413); Treatment with levothyroxine sodium (UKBB); Comparative height size at age 10 (UKBB) | LTA | chr6:31540429 |
| rs9385269 | Qualifications: college or university degree (UKBB); Years of educational attainment (PMID = 27225129); Leg fat percentage right (UKBB); Body fat percentage (UKBB); Time spent using computer (UKBB); Leg fat percentage left (UKBB); Trunk fat percentage (UKBB); Leg fat mass right (UKBB); Leg fat mass left (UKBB); Qualifications: none (UKBB); Arm fat percentage left (UKBB); Years of educational attainment in females (UKBB); Body mass index (UKBB) | RP11-436D23.1 | chr6:98547979 |
| rs2328887 | Self-reported malabsorption or coeliac disease (UKBB); Primary sclerosing cholangitis (PMID = 27992413); Intestinal malabsorption (UKBB); Mean corpuscular hemoglobin (PMID = 27863252); Mean corpuscular volume (PMID = 27863252); Platelet distribution width (PMID = 27863252); Reticulocyte fraction of red cells (PMID = 27863252); Mean corpuscular hemoglobin concentration (PMID = 27863252); Reticulocyte count (PMID = 27863252); White blood cell count (PMID = 27863252) | CARMIL1 | chr6:25430149 |
| rs2533273 | Average weekly beer plus cider intake (UKBB); Mineral and other dietary supplements: fish oil (UKBB) | DPP6 | chr7:153485282 |
| rs7808471 | NA |  |  |
| rs11772627 | Body mass index (UKBB); Arm fat percentage left (UKBB); Arm fat percentage right (UKBB); Arm fat mass left (UKBB); Qualifications: college or university degree (UKBB); Hip circumference (UKBB); Whole body fat mass (UKBB); Weight (UKBB); Trunk fat mass (UKBB) | MAD1L1 | chr7:2109821 |
| rs7829800 | NA |  |  |
| rs10740991 | Leg fat mass right (UKBB); Leg fat percentage right (UKBB); Waist circumference (UKBB); Body mass index (UKBB); Body fat percentage (UKBB); Weight (UKBB); Trunk fat percentage (UKBB); Arm predicted mass right (UKBB); Arm fat mass left (UKBB); Hip circumference (UKBB) | DNAJC1 | chr10:22058137 |
| rs7916868 | Body mass index (UKBB); Mean platelet volume (PMID = 27863252); Height (UKBB); Platelet count (PMID = 27863252); Platelet distribution width (PMID = 27863252); Fibrinogen levels (PMID = 26561523); Years of educational attainment (PMID = 27225129); Age completed full time education (UKBB) | JMJD1C | chr10:64988931 |
| rs893856 | NA |  |  |
| rs10896126 | NA |  |  |
| rs11037497 | Body mass index (UKBB); Whole body fat mass (UKBB); Comparative height size at age 10 (UKBB) | RP11-472I20.4 | chr11:43622423 |
| rs1622515 | Qualifications: college or university degree (UKBB); Qualifications: A levels or as levels or equivalent (UKBB); Qualifications: none (UKBB | NA | chr11:95523433 |
| rs3764002 | Leg fat percentage left (UKBB); Leg fat percentage right (UKBB); Sitting height (UKBB); Body mass index (UKBB); Body fat percentage (UKBB); Impedance of leg left (UKBB); Trunk fat percentage (UKBB); Arm fat percentage right (UKBB); Trunk fat mass (UKBB); Waist circumference (UKBB); Whole body fat mass (UKBB) | WSCD2 | chr12:108618630 |
| rs4140799 | Neuroticism score (UKBB); Sensitivity or hurt feelings (UKBB) | SIPA1L1 | chr14:72170969 |
| rs34162196 | NA |  |  |
| rs10129747 | NA |  |  |
| rs1797235 | NA |  |  |
| rs11632215 | NA |  |  |
| rs862227 | NA |  |  |
| rs1582322 | NA |  |  |
| rs62084586 | Monocyte percentage of white cells (PMID = 27863252); Granulocyte percentage of myeloid white cells (PMID = 27863252) | SUPT4H1 | chr17:56419228 |
| rs8081370 | NA |  |  |
| rs4800488 | Body mass index (UKBB); Weight (UKBB); Whole body fat mass (UKBB); Trunk fat mass (UKBB); Arm predicted mass left (UKBB); Waist circumference (UKBB); Hip circumference (UKBB); Leg predicted mass left (UKBB); Body fat percentage (UKBB); Height (UKBB) | NPC1 | chr18:21117571 |
| rs17175518 | Body mass index (UKBB); Whole body water mass (UKBB); Leg predicted mass left (UKBB); Trunk predicted mass (UKBB); Arm predicted mass left (UKBB); Weight (UKBB); Impedance of whole body (UKBB); Hip circumference (UKBB); Whole body fat mass (UKBB) | RP11-795H16.2 | chr18:57850583 |
| rs11152349 | NA |  |  |
| rs429358 | Body mass index (UKBB); Weight (UKBB); Trunk fat percentage (UKBB); Trunk fat mass (UKBB); Arm fat mass left (UKBB); Arm fat mass right (UKBB); Whole body fat mass (UKBB); Body fat percentage (UKBB); Leg fat mass right (UKBB); Leg fat percentage right (UKBB); Leg fat percentage left (UKBB) | APOE | chr19:45411941 |

UKBB: UK Biobank.

**Supplementary Table 3 |** Characteristics of SNPs extracted from exposure (dried fruit intake) GWAS statistical summary data.

| **SNP** | **Chromosome: Position** | **EA** | **OA** | **EAF** | ***F*-Statistic** | **Association with exposure** | |
| --- | --- | --- | --- | --- | --- | --- | --- |
|  |  |  |  |  |  | **Beta (SE)** | ***P*-value** |
| rs261809 | 1:241054465 | G | A | 0.5406 | 19.4224 | -0.0096(0.0017) | 9.80E-09 |
| rs11586016 | 1:44031793 | C | G | 0.3710 | 19.2087 | 0.0099(0.0017) | 1.10E-08 |
| rs12137234 | 1:72270797 | T | C | 0.3038 | 18.5802 | 0.0102(0.0018) | 2.80E-08 |
| rs72720396 | 1:91191582 | G | A | 0.2292 | 19.4555 | 0.0114(0.0020) | 8.70E-09 |
| rs11811826 | 1:204603861 | A | T | 0.2242 | 25.6372 | 0.0132(0.0020) | 4.40E-11 |
| rs3101339 | 1:72748669 | C | A | 0.6033 | 41.0534 | 0.0143(0.0017) | 6.20E-17 |
| rs75641275 | 1:98327133 | C | A | 0.1434 | 20.7769 | -0.0142(0.0024) | 2.90E-09 |
| rs7582086 | 2:60231826 | T | G | 0.4683 | 19.4714 | -0.0096(0.0017) | 8.80E-09 |
| rs7599488 | 2:60718347 | T | C | 0.4264 | 22.3821 | -0.0104(0.0017) | 6.70E-10 |
| rs4149513 | 2:101022726 | A | G | 0.4935 | 29.0129 | 0.0117(0.0017) | 2.20E-12 |
| rs17184707 | 2:166183577 | T | C | 0.2128 | 18.4883 | -0.0114(0.0020) | 2.10E-08 |
| rs4269101 | 3:18763543 | G | T | 0.7189 | 32.5084 | -0.0138(0.0019) | 1.10E-13 |
| rs11720884 | 3:43941406 | G | A | 0.2501 | 19.7760 | 0.0112(0.0019) | 7.60E-09 |
| rs57499472 | 3:147239337 | C | T | 0.4041 | 19.9591 | 0.0099(0.0017) | 8.10E-09 |
| rs10026792 | 4:2862190 | A | G | 0.2904 | 20.4509 | 0.0108(0.0018) | 3.90E-09 |
| rs1648404 | 4:37175523 | T | C | 0.4761 | 18.6549 | 0.0094(0.0017) | 1.80E-08 |
| rs746868 | 6:31540429 | G | C | 0.6147 | 33.2782 | -0.0129(0.0017) | 5.20E-14 |
| rs9385269 | 6:98547979 | T | C | 0.5246 | 30.6365 | 0.0121(0.0017) | 7.20E-13 |
| rs2328887 | 6:25430149 | C | T | 0.8995 | 27.3877 | 0.0189(0.0028) | 8.80E-12 |
| rs2533273 | 7:153485282 | A | C | 0.4845 | 20.5521 | -0.0099(0.0017) | 3.90E-09 |
| rs7808471 | 7:132716502 | C | T | 0.3221 | 24.5149 | -0.0115(0.0018) | 1.10E-10 |
| rs11772627 | 7:2109821 | C | G | 0.1820 | 42.2190 | 0.0183(0.0022) | 3.00E-17 |
| rs7829800 | 8:144258705 | G | A | 0.6710 | 20.3205 | -0.0104(0.0018) | 5.10E-09 |
| rs10740991 | 10:22058137 | C | G | 0.7176 | 47.9013 | 0.0167(0.0019) | 2.00E-19 |
| rs7916868 | 10:64988931 | T | A | 0.5035 | 19.4648 | 0.0096(0.0017) | 9.10E-09 |
| rs893856 | 10:126723567 | A | G | 0.1490 | 19.0933 | -0.0134(0.0023) | 1.30E-08 |
| rs10896126 | 11:66292908 | G | A | 0.3036 | 40.1780 | -0.0150(0.0018) | 1.60E-16 |
| rs11037497 | 11:43622423 | C | G | 0.4462 | 22.7178 | 0.0104(0.0017) | 5.70E-10 |
| rs1622515 | 11:95523433 | G | A | 0.4847 | 20.7219 | 0.0099(0.0017) | 2.90E-09 |
| rs3764002 | 12:108618630 | T | C | 0.2614 | 28.0431 | 0.0131(0.0019) | 5.10E-12 |
| rs4140799 | 14:72170969 | A | G | 0.5319 | 18.7828 | 0.0095(0.0017) | 1.80E-08 |
| rs34162196 | 14:22038125 | T | C | 0.1010 | 38.3066 | -0.0224(0.0028) | 7.10E-16 |
| rs10129747 | 14:77433198 | G | A | 0.5303 | 18.4045 | 0.0094(0.0017) | 2.60E-08 |
| rs1797235 | 15:47821612 | C | G | 0.3746 | 19.8446 | -0.0100(0.0017) | 8.90E-09 |
| rs11632215 | 15:45319982 | C | A | 0.1202 | 17.8421 | -0.0141(0.0026) | 4.40E-08 |
| rs862227 | 16:73602926 | G | A | 0.4583 | 17.5896 | -0.0092(0.0017) | 4.30E-08 |
| rs1582322 | 16:52105988 | G | A | 0.6048 | 19.9352 | 0.0099(0.0017) | 6.80E-09 |
| rs62084586 | 17:56419228 | C | T | 0.1657 | 20.9259 | 0.0134(0.0023) | 3.20E-09 |
| rs8081370 | 17:1373612 | T | C | 0.9102 | 19.1463 | -0.0167(0.0029) | 1.40E-08 |
| rs4800488 | 18:21117571 | A | C | 0.4899 | 30.2736 | 0.0120(0.0017) | 7.70E-13 |
| rs17175518 | 18:57850583 | A | C | 0.2328 | 19.9107 | 0.0115(0.002) | 5.90E-09 |
| rs11152349 | 18:60233646 | A | G | 0.3029 | 17.4989 | 0.0099(0.0018) | 4.90E-08 |
| rs429358 | 19:45411941 | C | T | 0.1542 | 43.7683 | 0.0199(0.0023) | 6.70E-18 |

SNP: single nucleotide polymorphism; EA: effect Allele; OA: other Allele; EFA: effect allele frequency; SE: standard error.

**Supplementary Table 4** **|** Heterogeneity test between dried fruit intake and eleven site-specific cancers in validation datasets

| **Outcome and method** | **Cochran's Q test** | | |
| --- | --- | --- | --- |
|  | ***Q*** | ***Q*_df** | ***Q*-value** |
| **Oral cavity/pharyngeal cancer** |  |  |  |
| **MR-Egger** | 34.7661 | 35 | 0.4793 |
| **IVW** | 35.3736 | 36 | 0.4982 |
| **Lung cancer** |  |  |  |
| **MR-Egger** | 35.5444 | 37 | 0.5373 |
| **IVW** | 36.0699 | 38 | 0.559 |
| **Squamous cell lung cancer** |  |  |  |
| **MR-Egger** | 33.9782 | 37 | 0.6115 |
| **IVW** | 34.1948 | 38 | 0.6461 |
| **Breast cancer** |  |  |  |
| **MR-Egger** | 43.8285 | 39 | 0.2741 |
| **IVW** | 43.8746 | 40 | 0.3107 |
| **Ovarian cancer** |  |  |  |
| **MR-Egger** | 38.3483 | 37 | 0.4082 |
| **IVW** | 38.352 | 38 | 0.4535 |
| **Pancreatic cancer** |  |  |  |
| **MR-Egger** | 35.4883 | 37 | 0.54 |
| **IVW** | 36.0572 | 38 | 0.5596 |
| **Cervical cancer** |  |  |  |
| **MR-Egger** | 36.981 | 37 | 0.47 |
| **IVW** | 38.9733 | 38 | 0.4258 |
| **Lung adenocarcinoma** |  |  |  |
| **MR-Egger** | 18.8264 | 37 | 0.9943 |
| **IVW** | 19.2993 | 38 | 0.995 |
| **Endometrial cancer** |  |  |  |
| **MR-Egger** | 16.8063 | 18 | 0.5365 |
| **IVW** | 18.7238 | 19 | 0.4747 |
| **Thyroid cancer** |  |  |  |
| **MR-Egger** | 37.4723 | 37 | 0.4474 |
| **IVW** | 39.2568 | 38 | 0.4133 |
| **Prostate cancer** |  |  |  |
| **MR-Egger** | 49.9569 | 37 | 0.0757 |
| **IVW** | 50.1933 | 38 | 0.089 |
| **Bladder cancer** |  |  |  |
| **MR-Egger** | 18.1159 | 37 | 0.9961 |
| **IVW** | 18.3357 | 38 | 0.997 |
| **Brain cancer** |  |  |  |
| **MR-Egger** | 33.9753 | 37 | 0.6116 |
| **IVW** | 34.326 | 38 | 0.6401 |

*Q*: Cochran’s *Q* statistic; df: degrees of freedom.

**Supplementary** **Table 5** **|** Heterogeneity test between fresh fruit intake and eleven site-specific cancers

| **Outcome and method** | **Cochran's Q test** | | |  | **Multiplicative random effects** | | |
| --- | --- | --- | --- | --- | --- | --- | --- |
|  | ***Q*** | ***Q*_df** | ***Q*-value** |  | **Beta** | ***SE*** | ***P*-value** |
| **Oral cavity/pharyngeal cancer** |  |  |  |  | 1.2508 | 0.6474 | 0.0533 |
| **MR-Egger** | 162.5251 | 27 | 2.63E×10^-21^ |  |  |  |  |
| **IVW** | 163.7889 | 28 | 3.85E×10^-21^ |  |  |  |  |
| **Lung cancer** |  |  |  |  | -0.0548 | 0.3658 | 0.8809 |
| **MR-Egger** | 69.2788 | 48 | 0.0238 |  |  |  |  |
| **IVW** | 70.3383 | 49 | 0.0245 |  |  |  |  |
| **Squamous cell lung cancer** |  |  |  |  | -0.7179 | 0.5806 | 0.2163 |
| **MR-Egger** | 76.0175 | 48 | 0.0061 |  |  |  |  |
| **IVW** | 76.6874 | 49 | 0.0069 |  |  |  |  |
| **Breast cancer** |  |  |  |  | -0.4314 | 0.2063 | 0.0365 |
| **MR-Egger** | 71.4899 | 50 | 0.0248 |  |  |  |  |
| **IVW** | 72.7583 | 51 | 0.0244 |  |  |  |  |
| **Ovarian cancer** |  |  |  |  | -0.477 | 0.2874 | 0.0969 |
| **MR-Egger** | 85.2272 | 51 | 0.0019 |  |  |  |  |
| **IVW** | 86.3561 | 52 | 0.0019 |  |  |  |  |
| **Pancreatic cancer** |  |  |  |  |  |  |  |
| **MR-Egger** | 1.9155 | 4 | 0.7513 |  |  |  |  |
| **IVW** | 1.921 | 5 | 0.86 |  |  |  |  |
| **Cervical cancer** |  |  |  |  |  |  |  |
| **MR-Egger** | 43.4671 | 44 | 0.4943 |  |  |  |  |
| **IVW** | 43.6245 | 45 | 0.5303 |  |  |  |  |
| **Lung adenocarcinoma** |  |  |  |  |  |  |  |
| **MR-Egger** | 54.8896 | 48 | 0.2298 |  |  |  |  |
| **IVW** | 55.1148 | 49 | 0.2545 |  |  |  |  |
| **Endometrial cancer** |  |  |  |  |  |  |  |
| **MR-Egger** | 65.7544 | 51 | 0.0801 |  |  |  |  |
| **IVW** | 66.3885 | 52 | 0.0865 |  |  |  |  |
| **Thyroid cancer** |  |  |  |  |  |  |  |
| **MR-Egger** | 10.047 | 7 | 0.1859 |  |  |  |  |
| **IVW** | 10.0624 | 8 | 0.2607 |  |  |  |  |
| **Prostate cancer** |  |  |  |  | 0.2608 | 0.1933 | 0.1772 |
| **MR-Egger** | 103.6109 | 51 | 1.90E×10^-5^ |  |  |  |  |
| **IVW** | 106.4122 | 52 | 1.30E×10^-5^ |  |  |  |  |
| **Bladder cancer** |  |  |  |  |  |  |  |
| **MR-Egger** | 44.7579 | 45 | 0.4821 |  |  |  |  |
| **IVW** | 44.7579 | 46 | 0.5243 |  |  |  |  |
| **Brain cancer** |  |  |  |  | -0.0037 | 0.0021 | 0.0703 |
| **MR-Egger** | 69.6931 | 44 | 0.0081 |  |  |  |  |
| **IVW** | 70.9824 | 45 | 0.0081 |  |  |  |  |
